# A novel alkaline protease from alkaliphilic *Idiomarina* sp. C9-1 with potential application for eco-friendly enzymatic dehairing in the leather industry

**DOI:** 10.1038/s41598-018-34416-5

**Published:** 2018-11-07

**Authors:** Cheng Zhou, Hongliang Qin, Xiujuan Chen, Yan Zhang, Yanfen Xue, Yanhe Ma

**Affiliations:** 1 State Key Laboratory of Microbial Resources, Institute of Microbiology, Chinese Academy of Sciences, Beijing, 100101 China; 2 National Engineering Laboratory for Industrial Enzymes, Institute of Microbiology, Chinese Academy of Sciences, Beijing, 100101 China; 3 School of Pharmaceutical Sciences, Jiangnan University, Wuxi, 214122 China

## Abstract

Alkaline proteases have a myriad of potential applications in many industrial processes such as detergent, food and feed production, waste management and the leather industry. In this study, we isolated several alkaline protease producing bacteria from soda lake soil samples. A novel serine alkaline protease (AprA) gene from alkaliphilic *Idiomarina* sp. C9-1 was cloned and expressed in *Escherichia coli*. The purified AprA and its pre-peptidase C-terminal (PPC) domain-truncated enzyme (AprA-PPC) showed maximum activity at pH 10.5 and 60 °C, and were active and stable in a wide range of pH and temperature. Ca^2+^ significantly improved the thermostability and increased the optimal temperature to 70 °C. Furthermore, both AprA and AprA-PPC showed good tolerance to surfactants and oxidizing and reducing agents. We found that the PPC domain contributed to AprA activity, thermostability and surfactant tolerance. With casein as substrate, AprA and AprA-PPC showed the highest specific activity of 42567.1 U mg^−1^ and 99511.9 U mg^−1^, the *K*_*m*_ values of 3.76 mg ml^−1^ and 3.98 mg ml^−1^, and the *V*_*max*_ values of 57538.5 U mg^−1^ and 108722.1 U mg^−1^, respectively. Secreted expression of AprA-PPC in *Bacillus subtilis* after 48 h cultivation resulted in yield of 4935.5 U ml^−1^ with productivity of 102.8 U ml^−1^ h^−1^, which is the highest reported in literature to date. Without adding any lime or sodium sulfide, both of which are harmful pollutants, AprA-PPC was effective in dehairing cattle hide and skins of goat, pig and rabbit in 8–12 h without causing significant damage to hairs and grain surface. Our results suggest that AprA-PPC may have great potentials for ecofriendly dehairing of animal skins in the leather industry.

## Introduction

Proteases (EC 3.4.21-24 and 99) catalyze the hydrolysis of peptide bonds of proteins by the addition of water across peptide bonds, and are widely found in plants, animals and microorganisms^1^. Proteases are classified into various groups based on their active sites and catalytic mechanisms, such as serine protease, cysteine protease, aspartic protease, metalloprotease, threonine protease, glutamic protease, and asparagine protease^2,3^. To date, there are 259 different proteolytic enzyme families in the MEROPS peptidase database (http://merops.sanger.ac.uk/) grouped by amino acid sequence similarity. Microorganisms, especially bacteria and fungi, comprise the main source of proteases and have been studied extensively. Proteases not only play important roles in cellular metabolic processes but also harbor desired characteristics for industrial applications^3^. Proteases from microbes are important industrial enzymes and account for approximately 40–60% of the total enzyme sales worldwide^4^.

Alkaline proteases are active from neutral to alkaline pH, which makes them highly desired in industries such as detergent manufacturing, food and feed production, peptide synthesis, leather processing, photography, silk degumming, and waste management^3,5^. Notably, alkaline proteases account for approximately a quarter of the total worldwide enzyme sales^1^. Owing to their broad applications, many alkaline proteases from alkaliphilic microbes have been studied in the past decades, with the majority of them found in *Bacillus* strains^5^. Some of them have been well characterized^6–16^. In addition to *Bacillus* bacterial, alkaline proteases from several gram-negative bacteria such as *Pseudomonas*^17^, *Xanthomonas*^18^, and *Vibrio*^19^ have also been found. Moreover, fungi especially genus *Aspergillus*^20,21^, yeast^22,23^, and actinomycetes^24,25^ are also prolific sources for alkaline proteases. Most of these alkaline proteases are either serine proteases or metalloproteases^3^, and show optimal pH around 8–12 and optimal temperature around 40–65 °C. Some thermostable alkaline proteases from thermophilic alkaliphiles have also been reported^26–28^.

Other than the heavy use in laundry detergents as cleansing additives, alkaline proteases have become increasingly important in dehairing animal skin and hides in the leather industry^29^. The conventional dehairing process is performed with a saturated solution of lime and sodium sulfide, which is known for high chemical oxygen demand (COD), biological oxygen demand (BOD), total suspended solid (TSS), total dissolved solids (TDS) and sulfide. As a consequence, the conventional process has been estimated to contribute 60–70% of the total pollution load associated with leather industrial processing^29,30^. Furthermore, the extensive use of sulfide is harmful to the health of the workers^31^. In addition, chemical processing is notorious for damaging leather besides weak performance in dehairing. Therefore, the enzyme-based dehairing process using alkaline protease, which holds promise to substantially reduce or replace the conventional lime and sodium sulfide effluent in addition to an improvement of leather quality, is being intensively pursued as an eco-friendly and viable alternative^31,32^.

Despite the advantages and increasing interests and demands, compared with laundry detergent, only a few alkaline proteases have been reported for dehairing in leather industry. These alkaline proteases are mostly from *Bacillus*^4,30,33,34^, *Paenibacillus*^35^, along with *Vibrio*^31^, *Pseudomonas*^36,37^, and *Aspergillus*^38,39^. There are still significant limitations in exploiting these alkaline proteases for industrial used, which include high cost for manufacturing, instability over wide range of pH and temperature, low tolerance to chemical reagents in leather processing, poor performance on dehairing, and significant damage to collagen^31,34,36^. Therefore, the search and development of novel alkaline proteases with high activity and complementary properties suitable for leather processing remain a challenge^31^.

In the present study, we isolated alkaliphilic bacteria from a soda lake in Inner Mongolia, China. An alkaline protease gene (*aprA*) from one of the strains, *Idiomarina* sp. C9-1, was cloned and expressed in *Escherichia coli* and *Bacillus subtilis*. We purified and characterized the biochemical properties of the recombinant enzyme and evaluated its ability in dehairing cattle hide and skins of goat, pig and rabbit. Our results showed that several features of this alkaline protease made it a promising enzyme for leather processing industry. To our knowledge, this is the first study on alkaline protease from a microorganism of genus *Idiomarina*.

## Results

### Screening and identification of protease-producing strains

After two enrichment-cultivation cycles, 41 strains showing protease activity were isolated from a soda lake using isolation agar plate. Through secondary screening in the format of shaking flask fermentation, 16 strains showing relatively higher activity (>50 U ml^−1^) were selected for further characterization. Crude enzymes from all 16 strains showed maximum activity at 50–60 °C and pH10.0–11.0, and were stable at temperature below 60 °C. Five strains termed C9-1, 3A-1, B4-1, DA1-1 and N1 were selected for further activity assessment and strain identification.

To identify the isolated strains, we sequenced 16 S rRNA genes and blasted against sequence database. As shown in Table 1, the five strains showed 99% sequence identity to *Idiomarina* sp. ST3, *Pseudidiomarina* sp., *Halomonas campisalis*, *Vibrio metschnikovii*, and *Bacillus pseudofirmus*, respectively. Next, we obtained the crude enzymes and examined their activity at different temperature and pH. The crude enzymes from all five strains showed optimal temperature at 60 °C except for N1, whose optimal temperature was 50 °C. The crude enzyme from 3A-1 showed maximum activity at pH11.0, whereas that from C9-1 was most active at pH10.5. The crude enzyme from all other three strains had an optimal pH of 10.0. In addition, we tested the stability of the crude enzymes. The crude enzymes from C9-1, 3A-1 and DA1-1 were more stable than the enzymes from B4-1 and N1. All five crude enzymes had good tolerance to JFC-2 and Peregal-O which usually occurs in leather industrial processes.

**Table 1:** Characterization of crude proteases from the screened strains.

Strains	Optimal temperature (°C)	Optimal pH	Activity (U ml^−1^)	Half-life at 60 °C (min)	Relative activity to JFC-2 and Peregal-O	The highest similarity of 16 S rDNA
C9-1	60	10.5	75.8	150	85%, 75%	*Idiomarinaceae bacterium* (98%)
3A-1	60	11.0	98.7	120	65%, 75%	*Pseudidiomarina* sp. (99%)
B4-1	60	10.0	87.9	90	75%, 70%	*Halomonas campisalis* (99%)
DA1-1	60	10.0	120.3	120	72%, 67%	*Vibrio metschnikovii* (99%)
N1	50	10.0	52.6	50	90%, 82%	*Bacillus pseudofirmus* (99%)

Based on above enzymatic properties, strain C9-1 was chosen for protease gene cloning and further study. The 16 S rRNA gene of C9-1 showed the highest homology to that of *Idiomarina* sp. ST3 with 99% identity. Other homologies are from *Idiomarina* sp. OM6, *Idiomarina* sp. CF11-10, *I. maris* strain CF12-14, *Idiomarina* sp. C4, *Idiomarina* sp. CF12-14, *I. salinarum* strain ISL-52, and *I. seosinensis* strain T8-3M, with sequence identity of 99%, 98%, 98%, 98%, 98%, 95%, and 95%, respectively. Subsequent studies revealed that the C9-1 was a rod-shaped, aerobic, non-pigmented, gram-negative, moderately halophilic and alkaliphilic bacterium, which were similar to the reported *Idiomarina* strains. Therefore, we concluded that the C9-1 isolate belongs to genus *Idiomarina* and is assigned as *Idiomarina* sp. C9-1.

### Gene cloning and sequence analysis of the AprA protease

**Figure 1:**
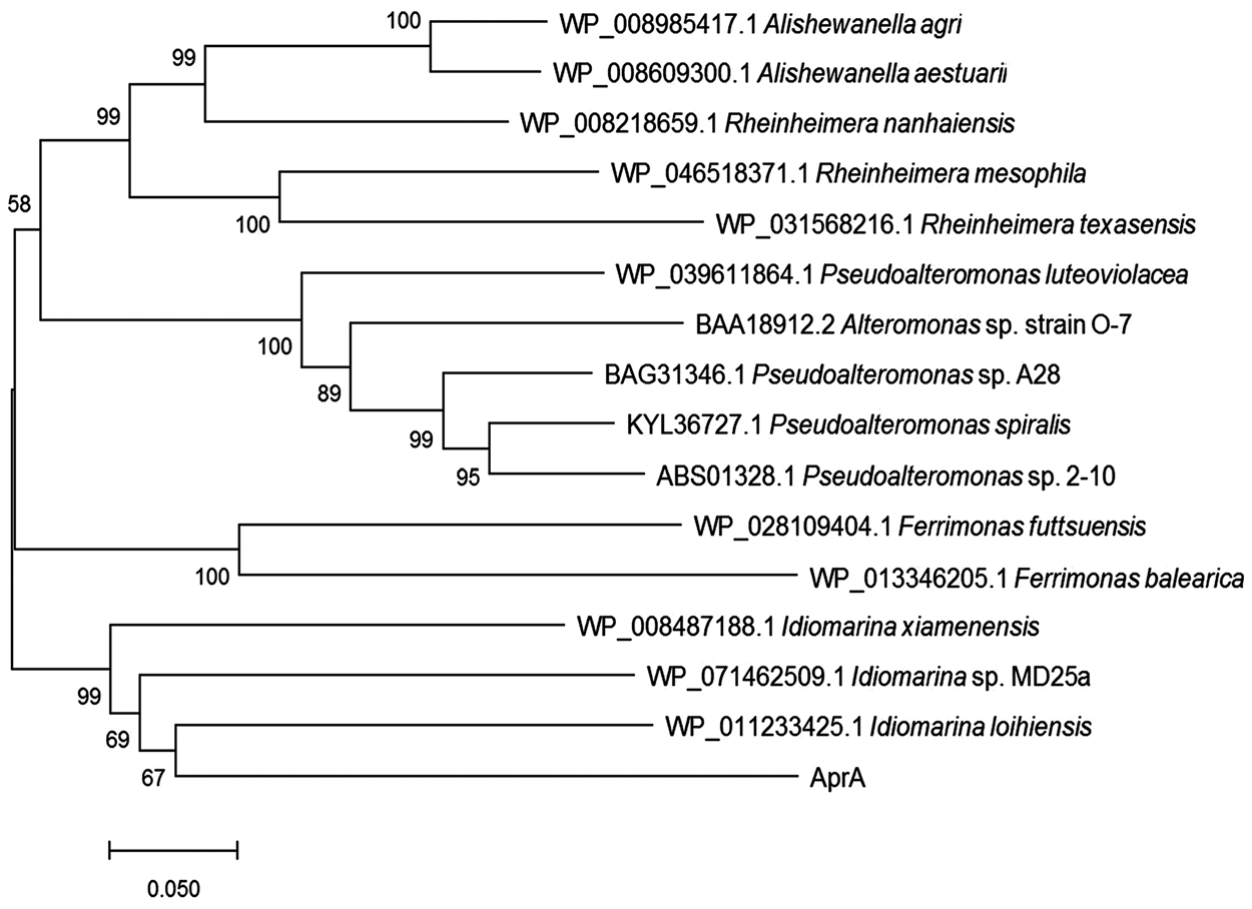
Phylogenetic analysis of AprA and other highly homologous proteases based on amino acid sequences. The GenBank accession No. for each serine protease is in front of the Latin name of each strain. The unrooted Neighbor-Joining tree was inferred using the MEGA 7.0 software. The percentage of replicate trees in which the associated taxa clustered together in the bootstrap test (1000 replicates) is shown next to the branches.

A genomic DNA library containing approximately 30,000 clones of *Idiomarina* bacterium C9-1 was successfully constructed. Of these, approximately 10,000 clones were screened and one positive clone with protease activity was obtained. An open reading frame (ORF) of 1,878 bp termed gene *aprA* was obtained by DNA sequencing. The *aprA* gene was predicted to encode a 625-amino-acid protein, which we termed protease AprA. The phylogenetic tree of AprA and some other highly homologous (identity ≥55%) proteases was generated using MEGA 7.0 based on amino acid sequence. As shown in Fig. 1, the AprA showed significant relatedness to peptidase from *Idiomarina* bacteria: they are localized at the same branch in the phylogenetic tree, indicating a common evolutionary origin.

The deduced protein sequence of AprA displayed high similarity to peptidase S8 from *Idiomarina* sp. MD25a (GenBank no. WP_071462509.1), *I. baltica* (GenBank no. WP_006955586.1), *Idiomarina* sp. T82-3 (GenBank no. KXS35504.1), *I. xiamenensis* (GenBank no. WP_008487188.1), *Alishewanella agri* (GenBank no. WP_008985417.1), *Idiomarina* sp. 28-8 (GenBank no. WP_034819033.1), and *I. loihiensis* (GenBank no. WP_011233425.1), with 63%, 63%, 63%, 61%, 60%, 59% and 59% identity, respectively. Additionally, AprA displayed high homology to the characterized serine proteases from *Pseudoalteromonas* sp. 2–10 (GenBank no. ABS01328.1)^40^, *Pseudoalteromonas* sp. A28 (GenBank no. BAG31346.1)^41^, *Alteromonas* sp. strain O-7 (GenBank no. BAA18912.2)^42^, *Stenotrophomonas maltophilia* 19580 (GenBank no. AEL88836.1)^43^ and *S. maltophilia* BBE11-1 (GenBank no. AGK29593.1)^44^ with 56%, 55%, 55%, 50% and 48% identity, respectively. Based on the phylogenetic tree and sequence identity analysis, we concluded that AprA is a serine protease that belongs to the S8A subfamily as classified by the MEROPS peptidase database (http://merops.sanger.ac.uk/).

**Figure 2:**
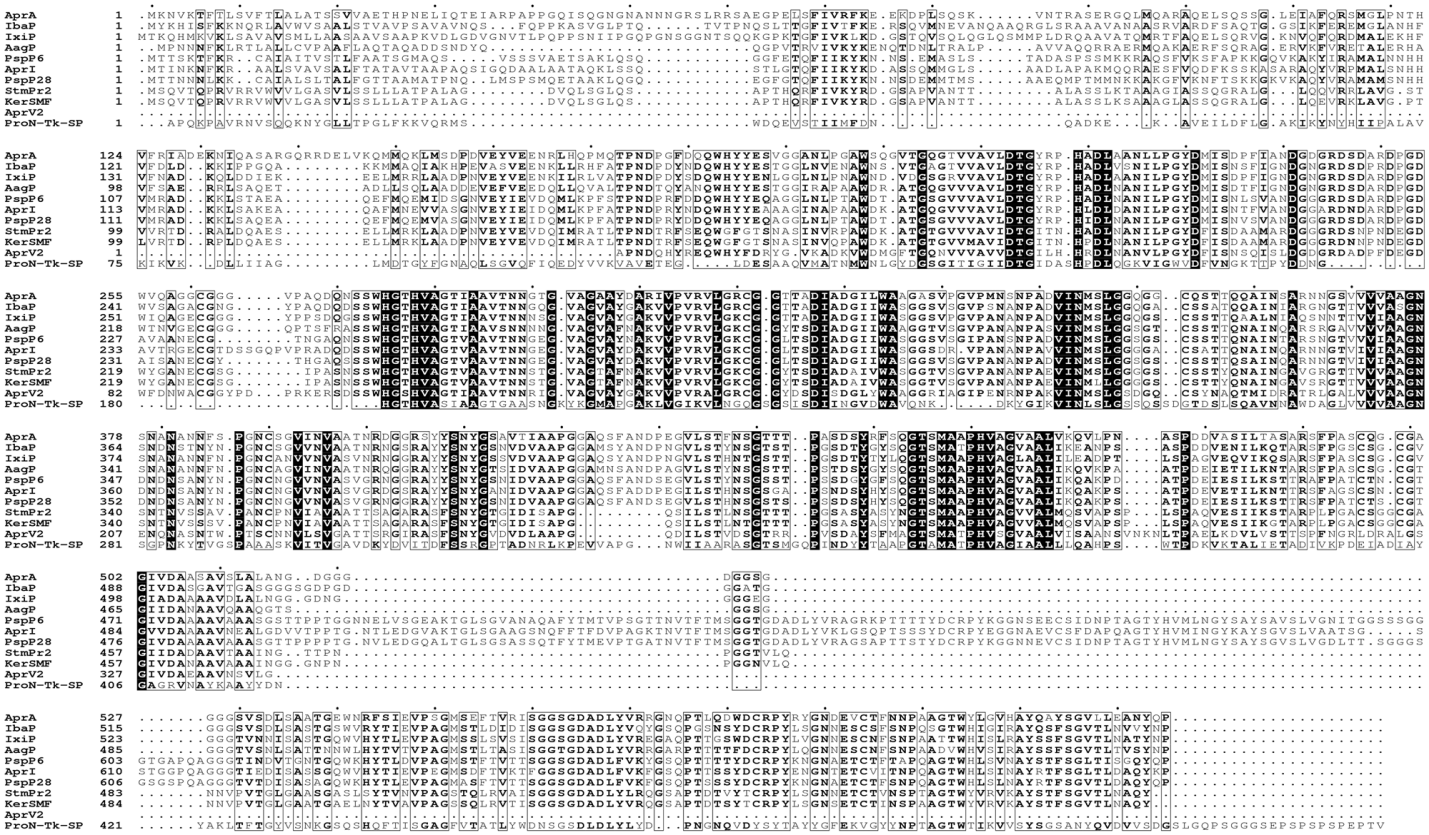
Multiple amino acid sequence alignment analysis. The proteases used were AprA, IbaP from *I. baltica* (GenBank No. WP_006955586.1), IxiP from *I. xiamenensis* (GenBank No. WP_008487188.1), AagP from *A. agri* (GenBank No. WP_008985417.1), PspP6 from *P*. sp. 2–10 (GenBank No. ABS01328.1), AprI from *P. piscicida* (GenBank No. BAA18912.2), PspP28 from *P*. sp. A28 (GenBank No. BAG31346.1), StmPr2 from *S. maltophilia* (GenBank No. AEL88836.1), KerSMF from *S. maltophilia* (GenBank No. AGK29593.1), AprV2 from *Dichelobacter nodosus* (PDB No. 3LPA_A) and Pro-TK-SP from *Thermococcus kodakaraensis* (PDB No. 3AFG_A). Strictly conserved residues are highlighted by black background and conservatively substituted residues are boxed. The vertical arrow shows the cleavage site of the signal peptide. The horizontal arrows show the N-terminal residues. The conserved catalytic residues are indicated by black circles and the PPC truncation site of AprA is indicated by black triangle. The residues in Ca-I and Ca-II binding sites of Pro-TK-SP were indicated by black and white pentagrams, respectively. The black circles indicated the residues of Ca-I binding site in AprV2, whereas white and black diamonds showed the residues of Ca-II and Ca-III binding site in AprV2, respectively. The figure was produced using ESPript 3.0.

**Figure 3:**
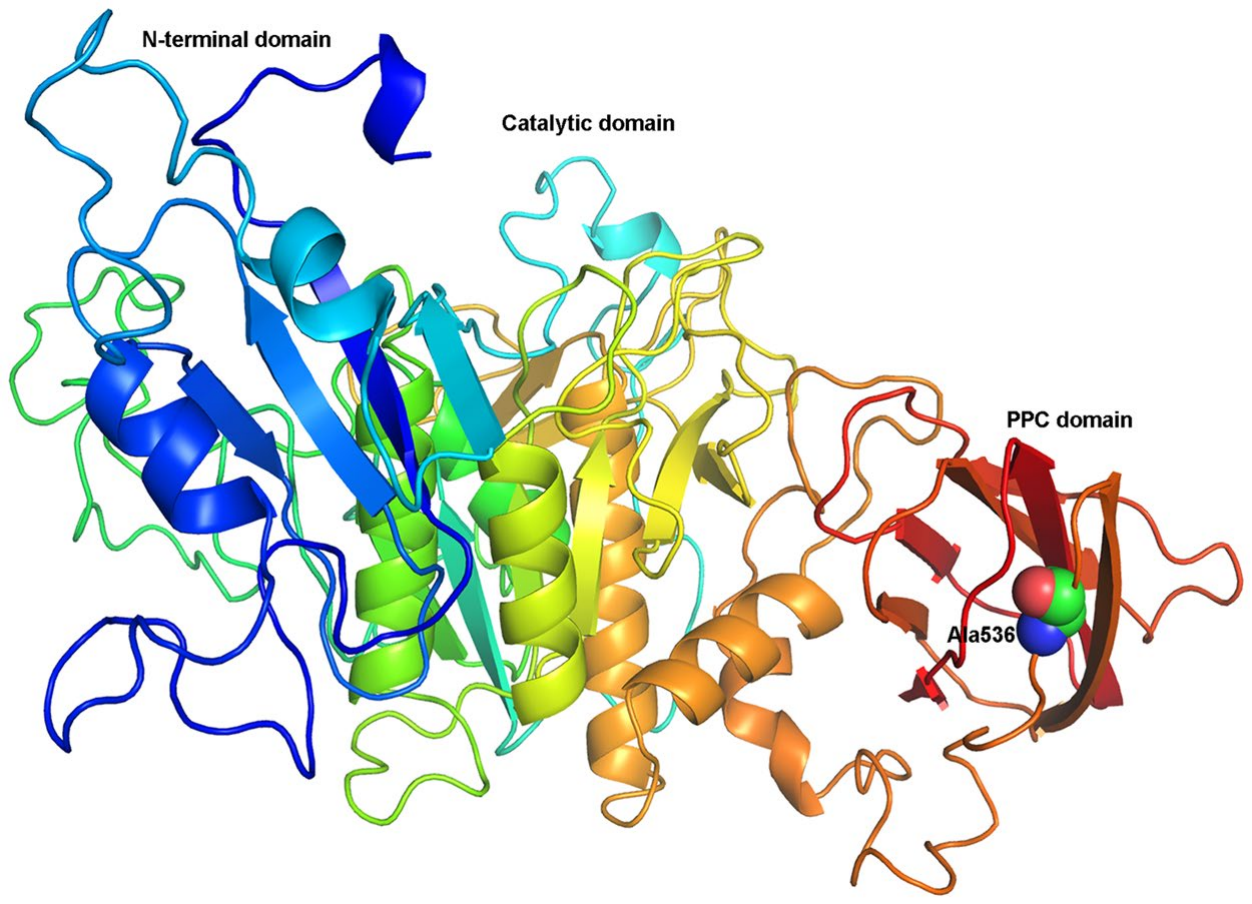
The modeled structure of protease AprA. Structure of serine protease Pro-TK-SP from *Thermococcus kodakaraensis* (PDB ID: 3AFG) was used as template. The PPC domain is represented in red. Truncation residue is displayed in spheres. Figure was developed using Pymol 0.99.

Based on the sequence alignment (Fig. 2), we proposed that the conserved catalytic triad of AprA could be Asp213-Thr-Gly (Asp213 as the active site), His275-Gly-Thr-His (His275 as the active site), and Gly-Thr-Ser456-Met-Ala-Ala-Pro (Ser456 as the active site). A typical amino-terminal signal peptide with 23 amino acids (Fig. 2) was identified using the SignalP 4.1 server (http://www.cbs.dtu.dk/services/SignalP/). Like some other peptidases, protease AprA also has an N-terminal domain (R59–Y186), a catalytic domain (G204–G502), and a pre-peptidase C-terminal (PPC) domain (E540–V608) (Fig. 2). The N-terminal sequencing and Nano-LC-MS/MS analysis showed that the N-terminal amino acid sequence of the mature AprA was AFQRSMGLPN (Fig. 2). This N-terminal sequence was different from other reported bacterial alkaline serine proteases including the 3 highly similar alkaline proteases from *Pseudoalteromonas* sp. 2–10, *Pseudoalteromonas* sp. A28 and *S. maltophilia* BBE11-1, which have a conserved five N-terminal amino acids of S/A/LTPND^40,41,44^. The modeled structure of full-length AprA was built (Fig. 3) based on the structure of a subtilisin-like serine protease (ProN-TK-SP) from *Thermococcus kodakaraensis* (PDB ID: 3AFG) which exhibited the best sequence coverage and about 32% total sequence identity with AprA in PDB database. The modeled AprA structure confirmed that there were also three functional domains and a linker comprising residues from 514 to 538 between the catalytic and the C-terminal domain. According to a previous report^45^, truncation close to a catalytic domain can result in the loss of activity. Therefore, Ala536 located inside the linker region but slightly distant from catalytic domain (Fig. 3) was mutated to generate the PPC domain truncation form of AprA.

The ProN-TK-SP contains two calcium ion binding sites located in PPC domain: Ca-I and Ca-II^46^. In the Ca-I binding site, the Ca^2+^ is coordinated with the side chain of Pro397, Ile400, Asp474 and Try475. In the Ca-II binding site, the Ca^2+^ is coordinated with the side chain of Asp460, Leu461, Asp462, Glu484 and Thr478. Despite the similarity in modeled structure, however, no similar calcium ion binding sites were found in AprA PPC domain (Fig. 2). Interestingly, a catalytic domain of a subtilisin-like protease AprV2 from *Dichelobacter nodosus* (PDB ID: 3LPA), which exhibiting a higher sequence identity (50%) to mature AprA, was also found in PDB database. In this structure, three calcium ion biding sites are showed^47^. The Ca^2+^ is coordinated with Asp4, Asp48, Val115, Asn118, Ile120 and Val122 in the Ca-I binding site, whereas Asp70, Gly71 and Asp73 are coordinated with Ca^2+^ in the Ca-II binding site. In the Ca-III binding site, Ca^2+^ is coordinated with Asp58, Asp68 and Asp75. As shown in Fig. 2, the corresponding conserved residues in Ca-II (Asp243, Gly244, Asp246) and Ca-III (Asp231, Asp241 and Asp248) binding sites were also found in the catalytic domain of AprA. Moreover, most catalytic domains of proteases that exhibit sequence similarity to AprA in the PDB database have 2–6 Ca^2+^ binding sites (data not shown). Based on above analysis, we speculated that there might be at least two Ca^2+^ binding sites in AprA catalytic domain.

### Heterologous expression and purification of recombinant AprA and AprA-PPC

**Figure 4:**
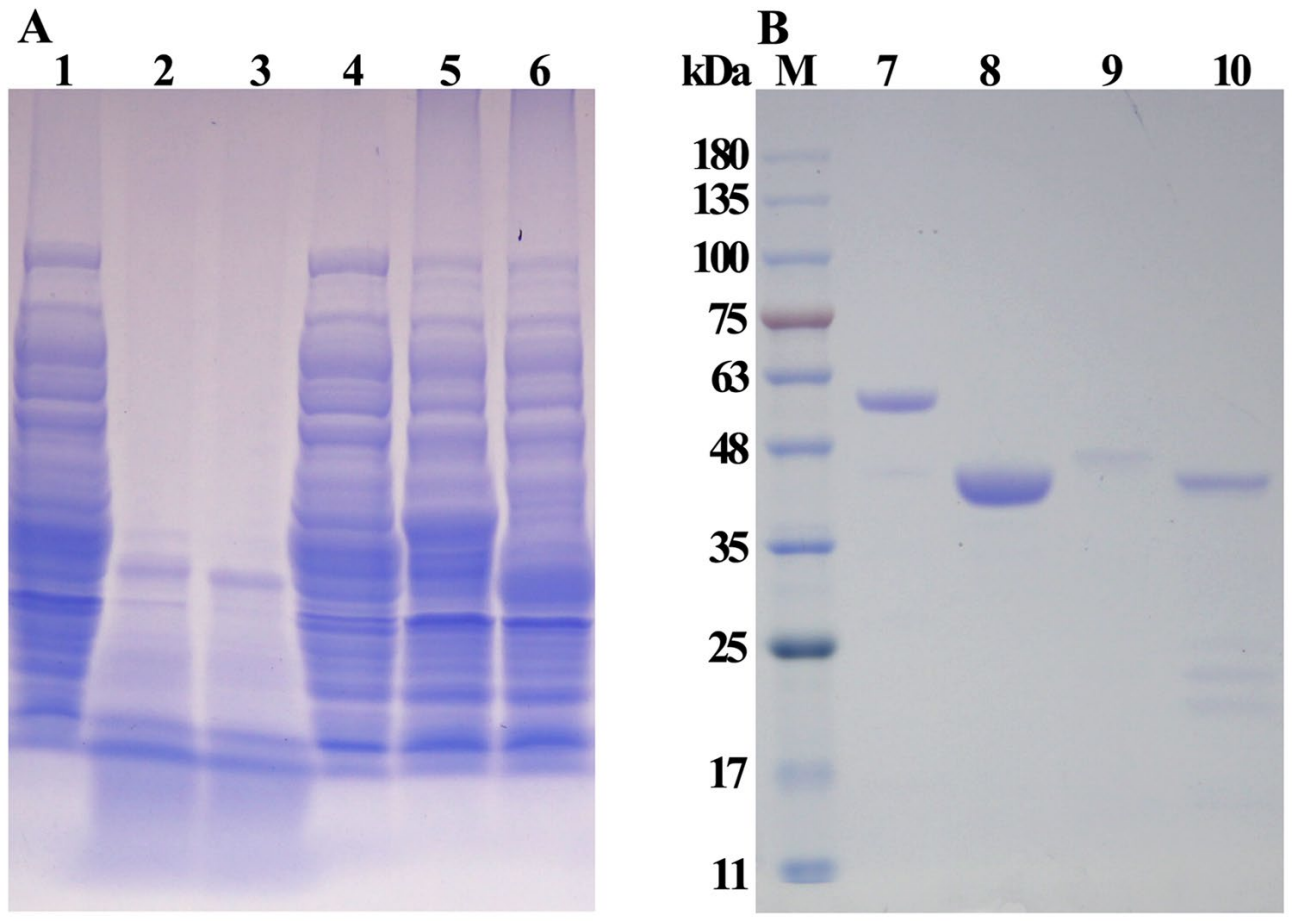
SDS-PAGE analysis of recombinant AprA and AprA-PPC. (**A**) The supernatant of crude extract from *E. coli* BL21 (DE3) PlysS expression strains. Lane 1, control containing pET28a without 10 mM PMSF; lane 2, AprA crude extract without 10 mM PMSF; lane 3, AprA-PPC crude extract without 10 mM PMSF; lane 4, control containing pET28a with PMSF; lane 5, AprA crude extract with PMSF; lane 6, AprA-PPC crude extract withPMSF. (**B**) The purified enzymes. Lane 7, purified recombinant AprA with 10 mM PMSF; lane 8, purified recombinant AprA-PPC with 10 mM PMSF; lane 9, purified recombinant AprA without PMSF; lane 10, purified recombinant AprA-PPC without PMSF. Lane M, molecular weight marker.

AprA and AprA-PPC, the truncated form of the AprA enzyme without the PPC domain, were each successfully expressed as soluble C-terminal His-tag proteins in *E. coli* BL21 (DE3) PlysS strains (Fig. 4A). The total activity of AprA and AprA-PPC by shake-flask cultivation reached approximately 732.6 and 779.5 U ml^−1^, respectively, after 20 h induction at 20 °C, with extracellular enzyme activity of only 2.7 and 2.9 U ml^−1^. However, the expression of AprA and AprA-PPC genes without signal peptide sequence was poor and almost no activity was detected after 20 h induction. These results indicated that the signal sequence might be important for expression of AprA in *E. coli* but might not contribute to its secretory expression, and also suggested that the PPC domain plays no roles in AprA secretion. Interestingly, protein degradation was observed in the cell lysis supernatant after boiling processing without addition of the protease inhibitor PMSF (Phenylmethylsulfonyl fluoride) (Fig. 4A), indicating that AprA and AprA-PPC exhibited proteolysis activity towards other proteins.

The recombinant AprA and AprA-PPC were purified to homogeneity with 10.2- and 8.1-fold purification, respectively. As shown in Fig. 4B, the mature AprA and AprA-PPC had a molecular mass of about 56 and 44 kDa, respectively, in line with the calculated molecular weight deduced from the amino acid sequence. Interestingly, the purified protein samples prepared without adding PMSF were partly degraded as shown on SDS-PAGE (Fig. 4B), indicating an autohydrolytic activity. Similar autohydrolysis phenomena have also been observed in other proteases^42,45,48–50^. However, unlike those proteases, autohydrolysis of AprA only occurred at high temperature (>60 °C), whereas it was stable at and below room temperature (data not shown).

### Effects of pH and temperature on the activity and stability of purified recombinant AprA and AprA-PPC

**Figure 5:**
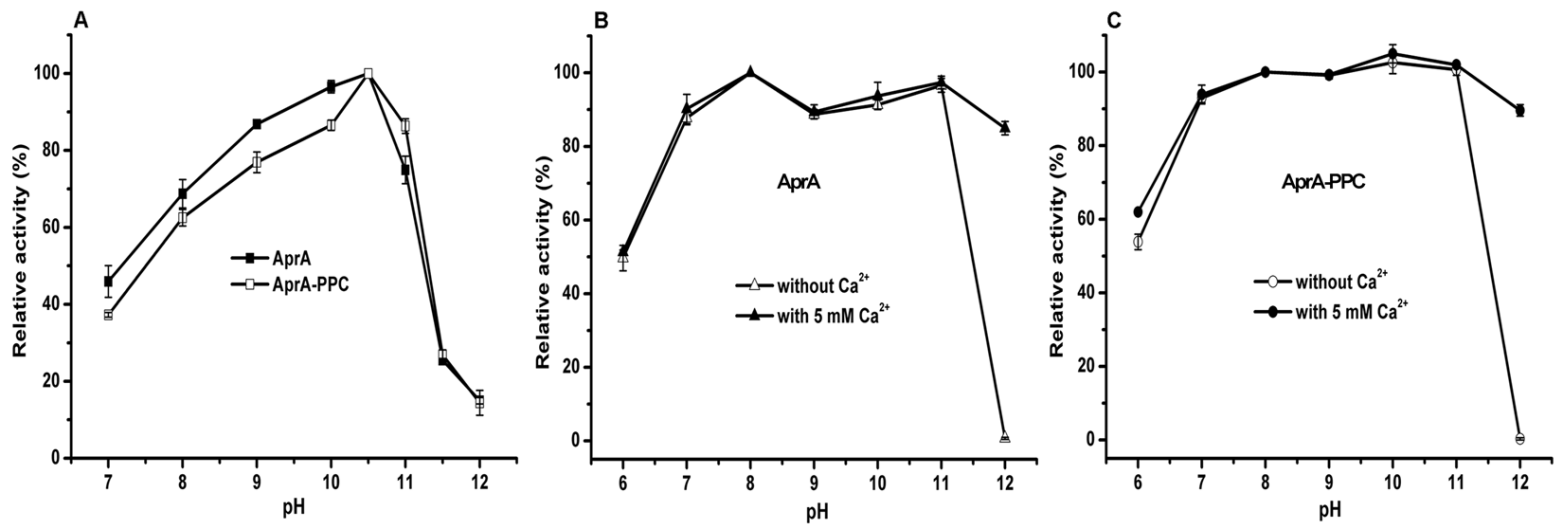
Effects of pH on AprA and AprA-PPC activity and stability with or without Ca^2+^. (**A**) The optimal pH for AprA activity (*filled square*) and AprA-PPC activity (*empty square*) without Ca^2+^; (**B**) pH stability of AprA with (*filled triangle*) or without (*empty triangle*) Ca^2+^; (**C**) pH stability of AprA-PPC with (*filled circle*) or without (*empty circle*) Ca^2+^. The measurements were performed in three independent experiments. Error bars represent standard deviations.

Ca^2+^ was previously considered to be important for the stability of some serine proteases^16,51^. Therefore, in our study, the effects of pH and temperature on recombinant AprA and AprA-PPC was determined using casein as substrate with or without adding 5.0 mM CaCl_2_. As shown in Fig. 5A, both AprA and AprA-PPC showed the optimal activity at pH 10.5, the same as that of the crude enzyme from the original *Idiomarina* sp. C9-1, but higher than that of similar alkaline proteases characterized before^40–44^. Over 60.0% of the activity was maintained at the pH range from 8.0 to 11.0. The activity declined rapidly at pH greater than 11.0. Ca^2+^ showed no significant influence on optimal reaction pH (data not shown). Both of AprA and AprA-PPC showed good stability at a wide pH range from 7.0 to 11.0 with or without CaCl_2_, and over 80.0% of the original enzyme activity was retained after 2 h at 37 °C (Fig. 5B,C). However, the stability of both enzymes at pH 12.0 was dramatically improved by the addition of 5.0 mM Ca^2+^ (90% of activity retained compared with complete inactivation). This indicates that Ca^2+^ is important for AprA and AprA-PPC to maintain their stability and activity under highly alkaline condition.

**Figure 6:**
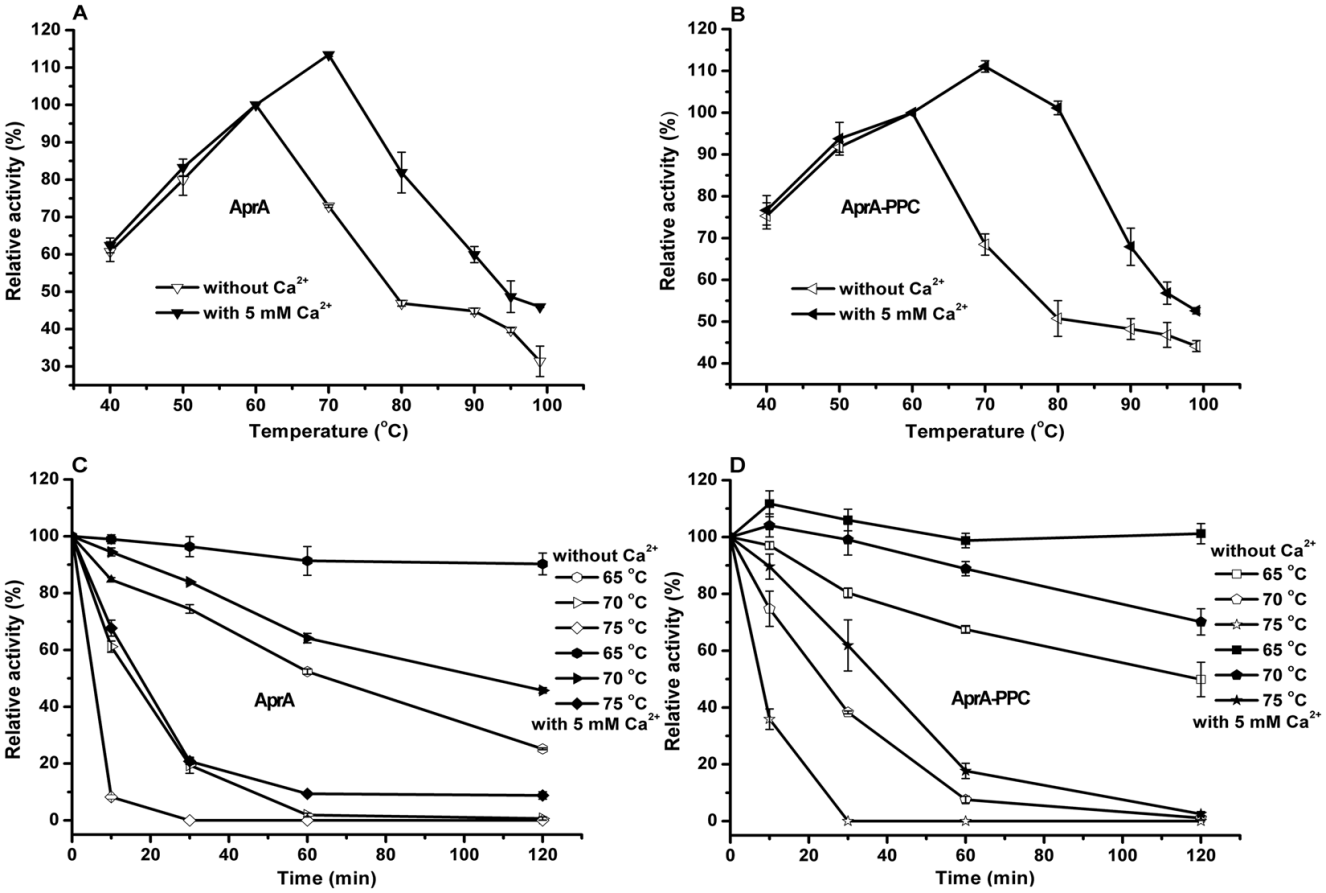
Effects of temperature on AprA and AprA-PPC activity and stability with or without Ca^2+^. (**A**) The optimal temperature for AprA activity; (**B**) The optimal temperature for AprA-PPC activity; (**C**) Thermal stability of AprA; (**D**) Thermal stability of AprA-PPC. Filled symbols indicated assay with 5 mM Ca^2+^, whereas the empty symbols indicated assay without Ca^2+^. The thermal stability was determined by assaying the residual activity of enzyme under standard condition after incubation at different temperature for various time intervals. The measurements were performed in three independent experiments. Error bars represent standard deviations.

Next we characterized the protease activity and thermostability of AprA and AprA-PPC under different temperatures. For both enzymes, more than 60.0% of the activity was maintained in the range of 40–70 °C (Fig. 6A,B). The optimal temperature for both enzymes was 60 °C without Ca^2+^, the same as that of crude enzyme from the original *Idiomarina* sp. C9-1, but higher than those for the five similar alkaline proteases characterized before. With 5.0 mM Ca^2+^, the optimal temperature increased to 70 °C. The upward shift of optimal temperature by Ca^2+^ has been reported for SPTC protease from *Trametes cingulate*^52^. In addition, adding Ca^2+^ also broadened the temperature range where more than 60.0% of activity was maintained. Furthermore, the relative activity of both enzymes in the presence of Ca^2+^ was obviously higher than that in the absence of Ca^2+^. The thermal stability assay showed that the half-life of AprA was 70 min at 65 °C, whereas only 21.0% and 8.0% of the original activity was retained after 30 min incubation at 70 °C and 10 min incubation at 75 °C, respectively (Fig. 6C). However, with 5.0 mM Ca^2+^, more than 90.0% of the original activity was retained after 70 min incubation at 65 °C, and approximately 65.0% and 68.0% of the original activity was retained after 60 min incubation at 70 °C and 10 min incubation at 75 °C, respectively (Fig. 6C). For AprA-PPC, the half-life was 120 min at 65 °C, whereas only 38.0% and 36.0% of the activity was retained after 30 min incubation at 70 °C and 10 min incubation at 75 °C, respectively (Fig. 6D). With 5.0 mM Ca^2+^, no activity loss was found after 120 min incubation at 65 °C, and approximately 70.0% and 60.0% of the original activity was retained after 120 min incubation at 70 °C and 30 min incubation at 75 °C, respectively (Fig. 6D). Meanwhile, the residual activity was slightly activated by approximately 11.7% and 4.1% upon incubation for 10 min at 65 °C and 70 °C, respectively. These results indicated that Ca^2+^ can improve not only the thermal activity of AprA and AprA-PPC but also the stability at high pH and temperature. In addition, the half-life of AprA-PPC was 120, 25, and 8 min at 65 °C, 70 °C and 75 °C, respectively, whereas that of AprA was 70, 15, and 4 min at the corresponding temperature. Therefore the thermal stability of AprA-PPC was better than that of AprA under the same condition regardless of the presence of Ca^2+^, which indicated that the PPC domain negatively affected the thermal stability of the AprA protease.

### Effects of metal ions and chemical reagents on the activity of the purified recombinant enzymes

The effect of metal ions and various chemicals on the activity of AprA and AprA-PPC was also evaluated and the results were shown in Table 2. For both AprA and AprA-PPC, Ca^2+^, Cu^2+^, Ba^2+^, Mn^2+^, Mg^2+^, and Co^2+^ increased whereas Ag^+^, Zn^2+^, Fe^2+^, Fe^3+^, Ni^2+^, Pb^2+^ and Hg^2+^ substantially decreased the enzyme activity. Other tested metal ions including K^+^, Na^+^, Al^3+^ and Li^+^ did not markedly affect the activity. The serine protease inhibitor PMSF completely inactivated both enzymes, indicating that AprA is a typical serine protease. The inhibiting effect of EDTA (Ethylene diamine tetraacetic acid) and EGTA (Ethylene glycol tetraacetic acid) gradually increased as their concentrations increase (Table 3). This might have occurred because of the chelation on Ca^2+^. The inhibitory effect of EDTA and EGTA suggested a requirement of metal ions for optimal activity. Some serine proteases have two Ca^2+^ binding sites, and Ca^2+^ removal by chelators would result in a significant decrease in thermal stability^53^. It is also interesting to note that AprA-PPC tolerated higher concentrations of EDTA and EGTA better than AprA.

**Table 2:** Effects of metal ions on activity of recombinant AprA and AprA-PPC.

Metal ions (5 mM)	Relative Activity (%)		Metal ions (5 mM)	Relative Activity (%)	
	AprA	AprA-PPC		AprA	AprA-PPC
Control	100.0	100.0	Fe^3+^	74.4 ± 1.7	67.0 ± 1.1
K^+^	101.4 ± 1.5	102.4 ± 3.1	Ba^2+^	107.8 ± 4.1	110.0 ± 2.9
Na^+^	100.2 ± 1.1	98.9 ± 2.5	Mn^2+^	119.7 ± 3.2	116.4 ± 3.1
Al^3+^	102.6 ± 1.6	99.7 ± 2.1	Co^2+^	112.0 ± 4.0	107.7 ± 2.7
Ca^2+^	115.4 ± 2.1	104.7 ± 1.8	Zn^2+^	66.2 ± 2.2	62.9 ± 1.9
Li^+^	100.9 ± 3.2	102.9 ± 3.1	Ag^+^	68.8 ± 3.1	71.7 ± 2.0
Cu^2+^	122.8 ± 3.8	107.8 ± 2.3	Fe^2+^	66.3 ± 1.4	63.2 ± 1.8
Mg^2+^	120.1 ± 2.4	115.2 ± 1.9	Ni^2+^	35.1 ± 1.0	33.6 ± 1.6
Hg^2+^	10.1 ± 0.6	9.5 ± 0.4	Pb^2+^	12.1 ± 0.7	11.7 ± 0.8

**Table 3:** Effect of detergents, oxidizing and reducing reagents and protease inhibitors on enzyme activity.

Detergent	Concentration	Relative activity of AprA (%)	Relative activity of AprA-PPC (%)
Control	—	100.0	100.0
Tween-20	1% (v/v)	92.6 ± 3.1	78.2 ± 1.0
Tween-40	1% (v/v)	92.5 ± 2.4	90.9 ± 1.4
Tween-80	1% (v/v)	82.5 ± 2.1	75.2 ± 0.9
Triton X-100	1% (v/v)	91.4 ± 3.0	83.9 ± 1.2
Triton X-155	1% (v/v)	99.1 ± 2.9	86.9 ± 1.7
H_2_O_2_	1% (v/v)	129.3 ± 4.2	121.5 ± 2.3
	2% (v/v)	126.1 ± 2.8	117.5 ± 2.1
	5% (v/v)	97.3 ± 1.9	84.7 ± 0.8
SDS	1% (w/v)	75.2 ± 2.6	71.9 ± 1.6
JFC-2	1% (v/v)	90.6 ± 1.2	103.8 ± 2.2
Peregal-O	1% (w/v)	88.6 ± 1.1	98.5 ± 2.0
β-Mercaptoethanol	1% (v/v)	87.6 ± 1.3	99.1 ± 1.4
PMSF	5 mM	0.0	0.0
EDTA	2 mM	88.9 ± 0.9	88.8 ± 1.2
	5 mM	42.4 ± 1.0	46.5 ± 0.4
	10 mM	13.6 ± 0.2	48.5 ± 0.7
EGTA	2 mM	85.4 ± 0.9	85.4 ± 1.1
	5 mM	37.5 ± 0.2	45.3 ± 0.8
	10 mM	7.4 ± 0.1	14.7 ± 0.5

Tolerance to surfactants, oxidizing and reducing agents are important for industrial application of proteases^29^. Our results showed that most of the tested reagents only slightly inhibited AprA activity except the surfactant SDS (Sodium dodecyl sulfate), which decreased the activity by approximately 25.0% (Table 3). SDS is an amphiphilic organosulphate that normally interacts with amino acid residues, causing protein unfolding therefore loss of enzymatic activity^16^. Similar effect was also found for AprA-PPC. However, AprA-PPC showed better tolerance to JFC-2, Peregal-O, β-Mercaptoetanol and high concentrations of EDTA and EGTA than AprA (Table 3). Both AprA and AprA-PPC showed good tolerance to H_2_O_2_ as only slight inhibition was found in 5.0% (v/v) H_2_O_2_. Interestingly, 1.0% and 2.0% H_2_O_2_ activated AprA activity approximately 29.3% and 26.1%, respectively, and that of AprA-PPC 21.5% and 17.5%, respectively (Table 3). The good tolerance to surfactants as well as oxidizing and reducing agents make AprA potential enzyme for industrial application.

### Substrate specificity and kinetic parameters of the purified recombinant enzymes

With respect to substrate specificity, both AprA and AprA-PPC showed specific activity towards casein, keratin, skim milk, BSA (Bull serum albumin) and gelatin to different extent. The activity of AprA towards these substrates, when normalized to casein as 100%, was 51.3% for keratin, 47.2% for skim milk, 17.3% for BSA and 8.0% for gelatin (Table 4). Conversely, only 2.5% activity on collagen was found. Almost the same results were obtained for AprA-PPC. AprA-PPC demonstrated the highest activity toward casein with 99511.9 U mg^−1^, which was higher than most previously reported alkaline proteases but lower than those from *B. clausii* I-52 (3.9 × 10^5^ U mg^−1^)^11^, *Termitomyces albuminosus* (1.8 × 10^5^ U mg^−1^)^54^ and *Vibrio metschnikovii* J1 (1.2 × 10^5^ U mg^−1^)^19^. High activity toward keratin (approximately 51447.7 U mg^−1^ for AprA-PPC) but low activity on gelatin suggested potential application in the leather industry for the dehairing. The *K*_*m*_ values for AprA and AprA-PPC on casein were 3.76 and 3.98 mg ml^−1^, respectively, and the *V*_*max*_ values were 57538.5 and 108722.1 U mg^−1^, respectively. As shown in Table 4, the *K*_*m*_ values for AprA and AprA-PPC showed no significant difference to the same substrate, indicating that the PPC domain truncation did not affect its binding to substrate.

**Table 4:** Substrate specificity and kinetic parameters of recombinant AprA and AprA-PPC.

Substrate	AprA			AprA-PPC		
	Relative activity (%)	*K*_*m*_ (mg ml^−1^)	*V*_*max*_ (U mg^−1^)	Relative activity (%)	*K*_*m*_ (mg ml^−1^)	*V*_*max*_ (U mg^−1^)
Casein	100.0	3.76 ± 0.08	57538.5 ± 785.4	100.0	3.98 ± 0.10	108722.1 ± 1801.2
BSA	17.3 ± 0.3	8.89 ± 0.21	11198.4 ± 223.3	21.7 ± 0.4	9.06 ± 0.24	25147.1 ± 348.1
Gelatin	8.0 ± 0.1	10.41 ± 0.29	5932.5 ± 97.5	8.4 ± 0.2	10.59 ± 0.31	9873.2 ± 131.6
Keratin	51.3 ± 1.2	5.38 ± 0.16	27921.1 ± 357.1	51.7 ± 1.4	5.25 ± 0.19	53272.6 ± 628.7
Skim milk	47.2 ± 1.0	6.02 ± 0.20	24378.3 ± 322.5	46.2 ± 1.1	6.12 ± 0.20	45196.8 ± 512.4
Collagen	2.5 ± 0.1	ND^*a*^	ND	2.6 ± 0.2	ND	ND

^a^ND means not determined.

### Efficient secreted expression in *Bacillus subtilis*

Eight different signal peptides (including the original signal peptide) were used for AprA-PPC expression in *B. subtilis* WB600 strain. All seven recombinant expression plasmids derived from pMA5 were successfully constructed. However, only two plasmids, pMA5-AprA-PPC2 and pMA5-AprA-PPC7, which contains signal peptide SP_*lipB*_ and SP_*amyl*_, respectively, were successfully transformed into *B. subtilis* WB600. We think that the other five signal peptides might not secrete AprA-PPC efficiently, resulting in accumulation of AprA-PPC, which in turn might be toxic to the *B. subtilis* WB600. The two viable recombinant strains, *B. subtilis* WB600A and *B. subtilis* WB600B containing SP_*lipB*_ and SP_*amyl*_ respectively, achieved an extracellular activity of 726.6 and 688.1 U ml^−1^ after 24 h incubation in LB medium. The secretory rates were measured to be 99.5% and 98.9% for WB600A and WB600B, respectively. Next, we tested six different media for recombinant protein expression in *B. subtilis* WB600A. As shown in Table 5, the activity and cell density in the medium containing glucose were the highest likely due to the fact that glucose is helpful to the cell growth, which then resulted in better AprA-PPC expression. The highest extracellular activity (3124.8 U ml^−1^) was obtained in SRG medium after 48 h cultivation. We also evaluated the effect of culture temperature on the expression of AprA-PPC. A higher activity (4935.5 U ml^−1^) was obtained when the strain was cultured at 30 °C after 48 h, which indicated that lower temperature was better for AprA-PPC expression in *B. subtilis*. The yield was also 65 times of the native enzyme activity from the original *Idiomarina* sp. C9-1.

**Table 5:** Extracellular protease activity in different expression medium.

Medium	Activity after 24 h (U ml^−1^)	Cell density (24 h OD_600_)	Activity after 48 h (U ml^−1^)	Cell density (48 h OD_600_)
LB	726.6 ± 10.1	3.8 ± 0.1	870.7 ± 11.4	4.5 ± 0.1
LBG	1530.2 ± 16.5	7.1 ± 0.2	2187.2 ± 26.3	9.1 ± 0.3
2 × LB	1358.0 ± 14.8	4.8 ± 0.1	1626.7 ± 16.2	7.3 ± 0.2
2 × LBG	1720.7 ± 20.5	7.2 ± 0.2	1943.7 ± 21.9	11.1 ± 0.4
SR	1738.7 ± 18.7	5.2 ± 0.2	1879.5 ± 18.5	8.5 ± 0.2
SRG	1952.4 ± 22.8	8.7 ± 0.3	3124.8 ± 28.3	11.5 ± 0.4
SRG-30 °C	2131.5 ± 25.1	7.0 ± 0.2	4935.5 ± 32.7	10.8 ± 0.3

### Animal skins dehairing evaluation by alkaline protease

**Figure 7:**
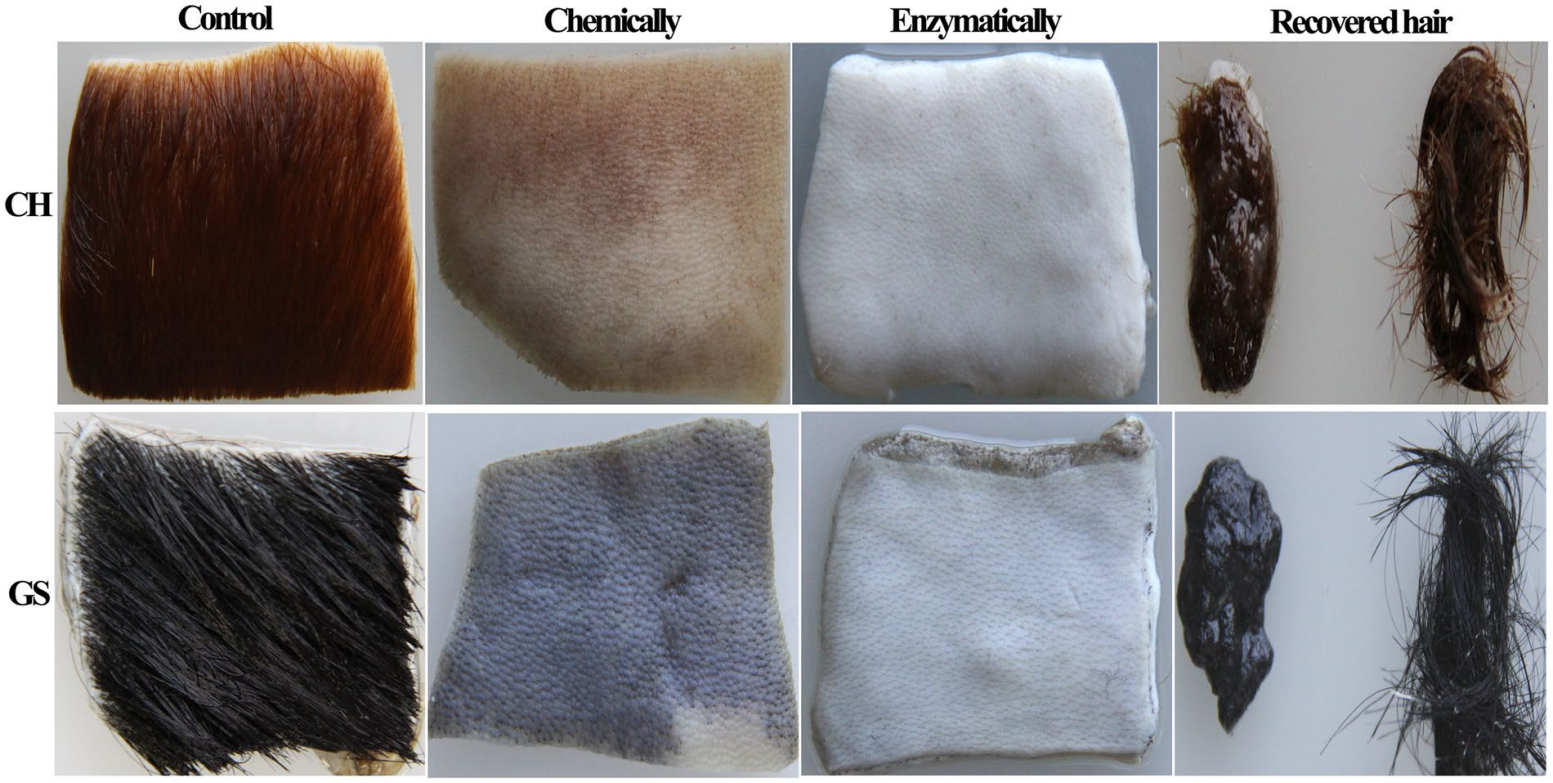
Dehairing effect by conventional chemical treatment versus enzymatic method with crude AprA-PPC. CH represents cattle hide; GS represents goat skin. The dehairing treatment was performed at 40 °C for 10–12 h. In the picture of recovered hair, chemical dehairing result is shown in the left, whereas enzymatic dehairing result is shown in the right.

**Figure 8:**
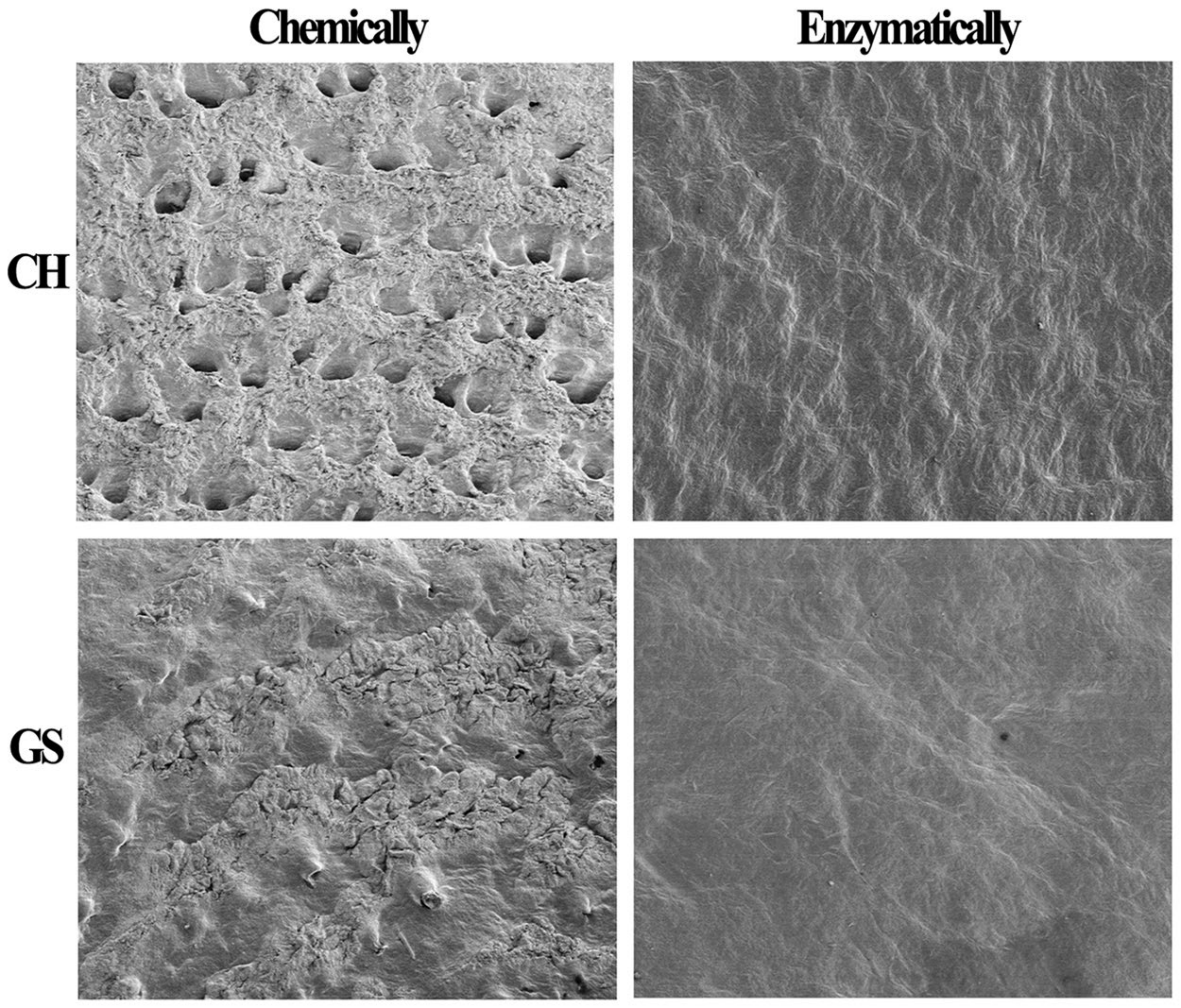
SEM images for the grain surface of chemically and enzymatically dehaired cattle hide and goat skin with ×30 magnification. CH represents cattle hide; GS represents goat skin.

**Figure 9:**
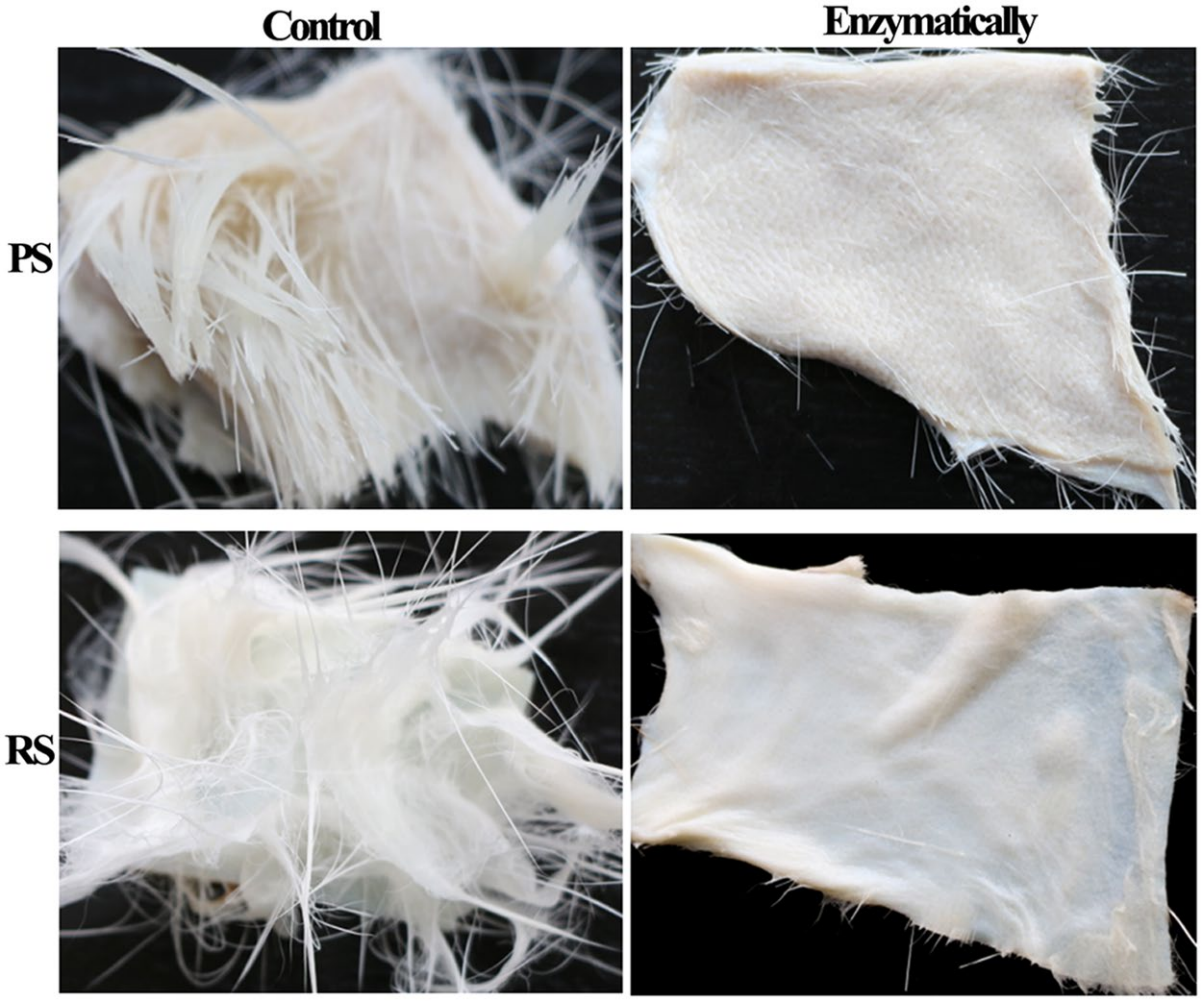
Enzymatic dehairing of pig and rabbit skins by crude AprA-PPC. PS represents pigskins, whereas RS represents rabbit skin. The dehairing treatment was performed at 40 °C for 8 h.

The dehairing ability of crude AprA-PPC from WB600A on cattle hide and goat skins were studied. As shown in Fig. 7, almost complete and uniform hair removal was observed on cattle hide and goat skins after 10–12 h treatment with alkaline protease AprA-PPC. Compared with chemical treatment, the enzyme treated skins were smoother, whiter, softer and slightly thinner. The recovered hair from enzymatic dehairing was intact owing to the absence of hair destructing sulfide, whereas the hair recovered from chemical dehairing was normally pulped with damage (Fig. 7). The SEM (Scanning electron microscope) was also used for morphological studies to compare the conventional chemical-treated with enzyme-treated skin samples. As shown in Fig. 8, the conventional pelt sample showed a white surface and several particles, which indicated the deposition of lime. In contrast, the enzyme-treated sample showed a clear surface without any deposition of foreign particles or grain damage. Although almost all the hair pores of conventional cattle hide sample were clear without unremoved hair root, there were many foreign particles around the hair pores (Fig. 8A). Chemical-treated goat skin sample showed the presence of unremoved hair root on the hair pore, indicating incomplete depilation (Fig. 8C). In contrast, enzyme-treated cattle hides and goat skins showed clean surface without any major deposition of foreign particles (Fig. 8B,D). We also tested the enzymatic dehairing effect of AprA-PPC on pig and rabbit skins. As shown in Fig. 9, almost complete hair removal was achieved after only 8 h of treatment with AprA-PPC. The effective dehairing without using damaging chemicals thus makes AprA-PPC an eco-friendly alkaline protease for leather industry.

## Discussion

Alkaliphilic microorganisms, derived mostly from alkaline environments such as soda lakes, constitute the main sources of alkaline proteases. In this study, we isolated several alkaline proteases producing alkaliphiles from soda lake in Inner Mongolia, China. Among them, *Idiomarina* sp. C9-1 produces an alkaline protease AprA with the highest activity and best tolerance to surfactant reagents. Genus *Idiomarina* comprises gram-negative, mesophilic and aerobic bacteria, and most reported strains were moderately halophilic or/and haloalkaliphilic^55^. To our knowledge, no protease from bacteria in this genus has been characterized. Our discovery of alkaline protease AprA from *Idiomarina* sp. C9-1 thus was the first case. AprA has low sequence similarity to other reported proteases (with the highest identity of 63.0% to directly submitted sequence in NCBI database), and is thus a novel alkaline serine protease.

AprA exhibits higher optimal pH (10.5) and better stability under alkaline conditions than many previously reported alkaline proteases from *Bacillus*^4,6,8,9,15,16,27,33,51,56–61^, *Vibrio*^19^, *Aspergillus*^21,62,63^, *Thermoactinomyces*^24,26^, *Streptomyces*^25^, *Thermus*^28^, *Pseudoalteromonas*^41^, *Alteromonas*^42^, *Stenotrophomonas*^44^, *Trametes*^52^, *Termitomyces*^54^, *Virgibacillus*^64^, *Caldicoprobacter*^65^, *Hirsutella*^66^, *Scopulariopsis*^67^, and *Penicillium*^68^ (Table S1). Furthermore, AprA also retains high activity and stability over a wide range of pH (7.0–11.5) and temperature (40–70 °C), several metal ions, surfactants and some oxidizing and reducing agents. These characteristics render AprA suitable for a variety of applications. In the leather manufacturing process, the pH and temperature varies depending on the region and season, and substantial amounts of Ca^2+^, Na^+^, and surfactants such as JFC-2 and Peregal-O are involved^69,70^. Therefore, AprA is especially suitable for leather manufacturing. AprA and AprA-PPC are also very stable in the presence of the oxidizing agent H_2_O_2_. In fact, their activities were increased by approximately 20.0% in the presence of 2.0% (v/v) H_2_O_2_ (Table 3). Tolerance to H_2_O_2_ has also been found for protease rBLAP from *B. lehensis*^16^ and AprX-SK37 from *Virgibacillus* sp. SK37^64^. Activation by low concentration of H_2_O_2_ has also been shown in protease SBcas3.3^71^ and SV1^72^. Generally, subtilases are inactivated by H_2_O_2_ because of methionine oxidization next to the catalytic serine, which then inhibits the formation of a tetrahedral intermediate during proteolysis^64,73^. AprA also has a methionine residue (Met457) that is located just after the catalytic serine (Ser456). However, the study of the oxidant-stable protease KP-43 showed that the oxidation of Met was not a fatal modification^74^. The structure analysis suggested that the rate of Met-oxidation in KP-43 was lower than those for other subtilases, probably due to the longer distance between the Met residue in the catalytic vicinity and the oxyanion hole^75^. Therefore, the oxidant-stable ability might depend on the intrinsic conformation and structural integrity^64^. In addition, the Met-oxidation in KP-43 also altered its substrate specificity. Therefore, we speculated that the activation of AprA by H_2_O_2_ might also be attributed to the effect of free radicals and perhydroxyl anions on its substrate in the catalytic center. Alkaline proteases exhibiting resistance to oxidizing agents and alkaline conditions are suitable for use as detergent additives^16^. Our study suggests that AprA could also serve as a promising candidate additive in the detergent industry.

Proteases are usually classified as a multi-domain enzyme. The PPC domain is generally found at the C-terminus of certain secreted bacterial peptidases such as some metalloprotease families and serine protease family S8, and usually is cleaved after secretion although prior to protease activation^76,77^. As shown in Fig. 2, the alignment of the C-terminal PPC domain from AprA and other homologies showed many hydrophobic residues such as valine, proline and the aromatic residue phenylalanine in the C-terminal domain. Previous structural homology modeling analyses showed that PPC domain comprised a parallel beta-sheets domain that was coupled to a catalytic domain through a loop linker^44–46^, which was also confirmed by structural modeling of AprA (Fig. 3). This might be a site prone to aggregation. The actual function of PPC domain remains unclear and there were only a few studies so far. For example, the PPC domain of the metalloprotease from *V. vulnificus* was essential for efficient attachment to insoluble substrates and erythrocyte membranes^78^. Proteases AprI and AprII displayed lower activity than the PPC domain truncated enzymes^42^. Studies on protease MCP-03 showed that the PPC domain decreased the catalytic activity but improved the thermostability^50^, whereas the β-jelly roll PPC domain stabilized protease Pro-TK-SP^46^. Additional research showed that the PPC domain truncation of protease HP70 and StmPr1 could improve the expression level of active protease in *E. coli* and the specific activity compared with the native enzyme^44,45^. In the current study, the specific activity of the PPC truncated enzyme, AprA-PPC (99511.9 U mg^−1^), was approximately 2.3-fold that of the parent enzyme AprA (42567.1 U mg^−1^), consistent with some studies^42,44,45,50^. However, opposite to ProN-TK-SP^46^ and MCP-03^50^, AprA lacking PPC domain shows enhanced thermostability (Fig. 5C,D). Moreover, the tolerance of AprA-PPC to JFC-2, Peregal-O, β-Mercaptoethanol and high concentrations of EDTA and EGTA is improved (Table 3). Combining previous and our current studies, we speculate that the PPC domain is related to the following functions: attachment to insoluble protein substrates (positive effect), thermal stability (positive or negative effect), catalytic efficiency and activity (negative effect), pH and surfactants stability (negative effect), and enzyme secretion (negative effect).

Ca^2+^ and other divalent ions such as Mn^2+^ and Mg^2+^ represent additional factors known to increase protease activity and thermostability^65,79^. Our current study showed that both AprA and AprA-PPC were activated by Ca^2+^, Mn^2+^, and Mg^2+^. These ions have been reported to activate proteases from organisms such as *Caldicoprobacter guelmensis*^75^, *Streptomyces koyangensis*^25^, *T. cingulate*^52^, and *Bacillus circulans*^56^. These studies suggest that the bivalent ions may stabilize enzyme structure, especially the active conformation at high temperature and protect the enzyme against thermal denaturation^5,47,52^. However, Cu^2+^, Ba^2+^, and Co^2+^ found to be inhibitory in these studies can stimulate AprA and AprA-PPC. The thermal denaturation experiments by GdnHCl (Guanidine Hydrochloride) inactivation also confirmed the results (Fig. S1). The residual activity of AprA and AprA-PPC after incubation at 60 °C for 10 min in the presence of Ca^2+^, Mg^2+^, Mn^2+^ or Cu^2+^ was higher than that in the absence of divalent metal ions, which indicated that these four divalent metal ions may stabilize the structure of AprA and AprA-PPC and protect the enzymes against thermal denaturation to various degrees. However, Co^2+^ and Ba^2+^ did not show the same effect on AprA and AprA-PPC. On the other hand, the activity of AprA and AprA-PPC was significantly but not completely inhibited by certain other bivalent ions including Hg^2+^, Pb^2+^, Ni^2+^, Fe^2+^, Zn^2+^, and the monatomic ion of Ag^+^. These toxic metallic ions might bind to particular organic ligands resulting in enzyme denaturation^52^. Notably, the inhibitory effect of heavy metallic ions such as Hg^2+^ and Pb^2+^ has been found to be a common phenomenon in many enzymes including proteases and is well documented in the literature. For example, Hg^2+^ is known to react with protein thiol groups such as histidine and tryptophan residues^80^. We also found that the thermostability of both AprA and AprA-PPC was also significantly improved by the addition of Ca^2+^. Similar results were also observed for several alkaline proteases from organisms such as *B. lehensis*^16^, *B. pumilus*^57^, *A. oryxae*^81^, *S. koyangensis*^25^, and *Conidiobolus brefeldianus*^79^. Ca^2+^ is considered to effect protease thermostability by strengthening the intramolecular interactions of the enzyme and by binding of Ca^2+^ to autolysis sites^79^. The result of thermal denaturation above in the presence or absence of Ca^2+^ also is in line with previous results.

Besides the enzymatic properties, high yield is also critical for industrial application. In the past years, species such as *E. coli*^27,43^, *B. subtilis*^58,82–85^, *Pichia pastoris*^12,62,86–88^, *Yarrowia lipolytica*^23^, *Saccharomyces cerevisiae*^89,90^ and *Zygosaccharomyces rouxi*^91^ have been used as hosts for the heterologous production of alkaline proteases (Table 6). However, very few alkaline protease genes from other genus such as *Aspergillus*^23,62,86,88,89,91^ and *Stenotrophomonas*^43^ have been successfully expressed. *B. subtilis* probably is more suitable for alkaline protease production in that the highest protease activity (5800.0 U ml^−1^ was obtained after 72 h incubation) has been reported^57^. In the present study, the AprA-PPC gene from *Idiomarina* sp. C9-1 was successfully expressed in *B. subtilis* and the extracellular activity of 4935.5 U ml^−1^ was obtained after 48 h cultivation with the highest productivity of 102.8 U ml^−1^ h^−1^ reported in literature to date (Table 6). This was the first report on heterologous expression of protease gene from genus *Idiomarina* in *B. subtilis*. Also, this yield was higher than most of other recombinant strains reported (Table 6). Moreover, this expression level can also be further improved by systematic optimization to a higher yield.

**Table 6:** Comparison of secretory expression of alkaline proteases in different strains.

Gene source and references	Expression strains	Expression vector	Culture time (h)	Activity (U ml^−1^)	Productivity (U ml^−1^ h^−1^)
*Idiomarina* sp.	*B. subtilis* WB600	pMA5	48	4935.5	102.8
*B. pumilus* ^56^	*B. subtilis* WB600	pSU	48	32.8	0.7
*B. subtilis* ^65^	*B. subtilis* DB100	pUB110	10	26.0	2.6
*B. pumilus* ^80^	*B. subtilis* DB430	pBSMuL2	72	5800.0	80.6
*Bacillus* sp.^79^	*B. subtilis* DB104	pAH101NC	24	30.0	1.3
*B. subtilis* ^57^	*B. subtilis* Bios11	pUB110	11	24.5	2.2
*A. oryzae* ^81^	*P. pastoris* GS115	pPIC9K	120	47.5	0.4
*B. stearothermophilus* ^12^	*P. pastoris* GS115	pGAPZαB	72	41.3	0.6
*A. nidulans* ^58^	*P. pastoris* X33	pPICZαA	48	1.0	<0.1
*B. cereus* ^82^	*P. pastoris* X33	pPICZαA	72	122.6	1.7
*A. sojae* ^83^	*P. pastoris* KM71	pPIC9K	72	400.4	5.6
*B. stearothermophilus* ^27^	*E. coli* XL1-Blue	pTrcHis	24	22	0.9
*S. maltophilia* ^43^	*E. coli* BL21-Gold	pMS470Δ8	20	60.0	3.0
Metagenome^45^	*E. coli* BL21-Gold	pMS470Δ8	28	27.0	1.0
*A. pullulans* ^23^	*Y. lipolytica* Po1h	pINA1317	96	49.5	0.5
*A. oryzae* ^84^	*S. cerevisiae* NA87-11A	pMA56	18	1.7	0.1
*Fusarium* sp.^85^	*S. cerevisiae* AH22R	pGAN1	72	15.1	0.2
*A. oryzae* ^86^	*Z. rouxii* ATCC13356	pZAP101A	168	166.1	1.0

Lime and sulfide constitute the main pollution generated during dehairing process in the leather industry. In recent years, enzymatic dehairing by protease have been reported^32,38,69^, however, many remains using lime and sulfide. Among these processes, enzymes carrying sparingly soluble kaolin or soluble silicates were used for lime and sulfide free enzymatic dehairing, which can still cause a significant increase in COD, BOD, TDS and TSS of the effluent^38,92^. Alkaline protease preparations specifically from organisms such as *V. metschnikovii*^31^, some *Bacillus* strains^30,93,94^, and *Pseudomonas fluorescens*^37^ have also been reported for lime and sulfide free dehairing. Nevertheless, these enzymes were generated directly from strain cultures with low activity. Alternatively, in the current study, the alkaline protease AprA-PPC with higher specificity and activity was efficiently expressed in *B. subtilis*, yielding high activity in culture medium. The crude enzyme was very effective in dehairing animal skins after 8–12 h treatment without any use of lime and sulfide. Also, the dehairing time was less than reported 12 to 24 h for different animal skins in the literature^4,30,37,38,95^, and only longer than the processes by a commercial protease^34^ and an alkaline protease from *B. subtilis* BLBc 11^93^ (6 h duration). A shorter dehairing process by alkaline protease not only mitigates damage to collagen^34^, but also reduces constraint of a high degree of control. In addition, the dehairing of animal skins by AprA-PPC yielded intact hair, which may represent a valuable byproduct. Further, approximately 70% of the waste from pretanning processes is resulted from hair rich in nitrogen^34^. Enzymatic dehairing with intact recovered hair would thereby significantly reduce COD in the process.

In summary, our present study is the first to characterize protease from genus *Idiomarina*, and the first to express the protease heterologously in *B. subtilis*. The alkaline protease AprA and its PPC domain truncated enzyme AprA-PPC from *Idiomarina* sp. C9-1 showed high activity and good stability over a wide range of pH and temperature, and also displayed excellent tolerance to some surfactants as well as oxidizing and reducing agents, which hold promises for broad industrial applications. Its performance in dehairing animal skins is advantageous over the chemical process, making it especially valuable in the leather industry. In addition, our study on the PPC domain provides new information to better understand the function of proteases across different species.

## Materials and Methods

### Alkaline protease-producing strain screening

The soil sample was collected from a soda lake with pH of 9.86 and salinity of 20%, in Hulunbuir of Inner Mongolia, China. The enrichment medium contained 5.0 g l^−1^ tryptone, 1.0 g l^−1^ yeast extract, 0.2 g l^−1^ MgSO_4_·7H_2_O, 1.0 g l^−1^ K_2_HPO_4_, 0.2 g l^−1^ CaCl_2_, and 30.0 g l^−1^ NaCl. The isolation medium was made from enrichment medium by replacing tryptone to skim milk and added 2% (w/v) agar. The fermentation medium was obtained from enrichment medium by changing the concentration of tryptone to 10.0 g l^−1^. All the medium were sterilized at 115 °C for 15 min and then 100.0 ml l^−1^ 10% (w/v) sterilized Na_2_CO_3_ solution was added to adjust the pH to 10.0 before use.

About 10.0 g soil sample was added into 100.0 ml enrichment medium and incubated at 37 °C with shaking for 48 h and then about 5.0 ml of the cultures were inoculated into 100.0 ml fresh enrichment medium and incubated under the same condition for the second enrichment culture. The twice enriched culture was gradient diluted and plated onto the isolation medium, and then was incubated at 37 °C for 24 h. The protease producing strain can form transparent zone and the strains with big transparent zone were chosen and inoculated into 50.0 ml fermentation medium and then incubated for 48 h at 37 °C with shaking for further crude enzyme activity evaluation.

The identification of the selected protease producing strains was performed by sequencing analysis of 16 S rRNA genes. The genomic DNA was extracted by the Bacterial DNA Kit (OMEGA Bio-tek, Norcross, USA). The primer pair of F (5′-AGAGTTTGATCCTGGCTCAG-3′) and R (5′-TACGGCTACCTTGTTACGACTT -3′) were used for 16 S rRNA gene amplification by polymerase chain reaction (PCR). The PCR program was as following: 1 cycle of 95 °C for 3 min; 30 cycles of 95 °C for 35 s, 56 °C for 30 s, and 72 °C for 1 min; and the final extension was performed at 72 °C for 10 min. The 16 S rRNA genes were sequenced by SinoGenoMax Co., Ltd. (Beijing, China).

### Strains, plasmids and materials for gene cloning and expression

The plasmids and bacteria strains used in this study for gene cloning and expression were listed in Table S2. Casein, skim milk, keratin, gelatin, collagen and bovine serum albumin (BSA) were from Sigma-Aldrich (St. Louis, MO, USA). All the enzymes for DNA manipulations were purchased from NEB (Dalian, Liaoning, China). Phenylmethanesulfonyl fluoride (PMSF), isopropyl-β-D-thiogalactopyranoside (IPTG), imidazole, ampicillin, and kanamycin were from Amresco Inc. (Solon, OH, USA). All other chemicals used in this study were of reagent grade.

### Gene cloning and expression plasmid construction of alkaline protease AprA

*Idiomarina* sp. C9-1, which was isolated from a soda lake in Hulunbuir of Inner Mongolia and exhibited high alkaline protease activity, was used as DNA source for the alkaline protease (AprA) gene cloning. *Idiomarina* sp. C9-1 was also preserved in the China General Microbiological Culture Collection Center (CGMCC 1.16117). The restriction enzyme *Sau*3A was employed to obtain randomly digested chromosomal fragments. These 3.0- to 8.0-kb fragments were recovered and purified by the Gel Extraction Kit (OMEGA Bio-tek) and ligated into the *Bam*HI digested pUC118 vector treated with alkaline phosphatase. The ligation product was then transformed into *E. coli* DH5α cells by electroporation transformation and plated onto the screen medium (pH 8.0) containing 5.0 g l^−1^ yeast extract, 10.0 g l^−1^ tryptone, 10.0 g l^−1^ NaCl, 10.0 g l^−1^ skim milk, 15.0 g l^−1^ agar and 60.0 μg ml^−1^ ampicillin. After incubation at 37 °C for 24 h, the colony with transparent zone was selected for the inserted fragment sequencing by SinoGenoMax Co., Ltd. (Beijing, China).

The AprA-encoding gene (*aprA*) was obtained by PCR using the primer pair F1 (5′-CATGCCATGGGCATGAAGAATGTTAAAACATT-3′, where the underline indicates the *Nco*I site) and R1 (5′-ACCGCTCGAGCGGCTGGTAATTTGCTTCAA-3′, where the underline indicates the *Xho*I site). The enzyme without pre-peptidase C-terminal (PPC) domain (AprA-PPC) encoding gene fragment was obtained by PCR using the primer pair F1 and R2 (5′-ACCGCTCGAGCGCAGATAAGTCCGACACGC-3′, where the underline indicates the *Xho*I site). The AprA and AprA-PPC encoding gene without the signal peptide sequence were also obtained by PCR using primer pair of F2 (5′-CATGCCATGGGCGAAACTCATCCTAACGAGCT-3′, where the underline indicates the *Nco*I site) and R1, and of F2 and R2, respectively. The following PCR programs were used: 1 cycle of 95 °C for 3 min; 30 cycles of 95 °C for 35 s, 56 °C for 35 s, and 72 °C for 90 s; a final extension at 72 °C for 10 min was followed. The PCR products were purified by the OMEGA Gel Extraction Kit and then digested with *Nco*I and *Xho*I for ligation to the pET28a vector which was cleaved with the same two restriction enzymes. The resultant recombinant plasmids were transformed into *E. coli* BL21 (DE3) PlysS competent cells for gene expression.

### Sequence analysis and tertiary structure modeling

The gene sequence of AprA was analyzed by the NCBI BLAST program. The signal peptide prediction was performed by the SignalP 4.1 server (http://www.cbs.dtu.dk/services/SignalP/). The alignment analysis of amino acid sequences was performed by ClustalX. The phylogenetic tree of protein sequences was constructed by MEGA 7.0 with neighbor-joining (NJ) method. The three dimensional (3D) structure was modeled by SWISS-MODEL online server (https://www.swissmodel.expasy.org/). Figure was developed by Pymol 0.99 software.

### Heterologous recombinant expression in *E. coli* and purification

The recombinant *E. coli* BL21 (DE3) PlysS strains were cultured in 0.5 l Luria-Bertani (LB) medium (10.0 g l^−1^ tryptone, 5.0 g l^−1^ yeast extract, 10.0 g l^−1^ NaCl) containing 60.0 μg ml^−1^ of kanamycin at 37 °C. IPTG was added with a final concentration of 0.1 mM when the OD_600_ reached 0.6. After continuous cultivation at 20 °C for 20 h, the cells were harvested by centrifugation at 8,000 × *g* for 10 min. The recombinant enzymes were purified by nickel-nitrilotriacetic acid (Ni-NTA) affinity chromatography and gel filtration chromatography. The harvested cells were washed with the Ni-NTA binding buffer^41^ and then were suspended in 40.0 ml binding buffer. After ultrasonication and centrifugation at 15,000 × *g* for 25 min at 4 °C, the supernatant was loaded onto a pre-equilibrated Ni-NTA column (Qiagen, Valencia, CA, USA) and flowed out by gravity. Then the column was washed with 15.0 ml binding buffer and 15.0 ml modified washing buffer (20.0 mM Tris-HCl buffer containing 0.5 M NaCl and 20.0 mM imidazole, pH 7.9). Ultimately, the enzyme was eluted with 2.5 ml of the Ni-NTA elution buffer^96^.

The protein solution obtained by Ni-NTA affinity chromatography was desalted using the desalting column (GE Healthcare Bio-Sciences AB, Uppsala, Sweden) with 20.0 mM Tris-HCl buffer (pH 7.5). Then the desalted protein solution was concentrated to 0.5 ml by an Amicon Ultra-15 centrifugal filter (Ultracel-30K) (Merk Millipore, Tullagreen, CORK IRELAND) and subsequently loaded onto a Superdex 75 10/300 GL column which was pre-equilibrated with the eluent buffer (50.0 mM Tris-HCl, 150.0 mM NaCl, pH 7.5). A flow rate of 0.4 ml min^−1^ was used and the desired protein fractions were collected. The collected protein solution was desalted with 20.0 mM sodium Tris-HCl buffer (pH 7.5).

The protein concentration was determined by the Quick Start Bradford protein assay (Bio-Rad, Hercules, CA, USA) with bovine serum albumin (BSA) (0.1–0.8 mg ml^−1^) as standard. The purity of the enzyme was assessed by 12.0% sodium dodecyl sulfate polyacrylamide gel electrophoresis (SDS-PAGE) analysis with or without 10 mM PMSF. The N-terminal amino acid sequence was determined by Nano-LC-MS/MS and N-terminal sequencing analysis. The Nano-LC separation was conducted using a Thermo Scientific™ EASY-nLC™ 1000 HPLC system (Waltham, MA, USA). All the separated peptide fractions were then analyzed using a Thermo Orbitrap Fusion mass spectrometer (Waltham, MA, USA). Data was acquired on the Orbitrap Fusion MS using a resolution of 120,000 (@ 200 m/z) for full MS scans followed by HCD fragmentation and detection of the fragment ions in the Orbitrap. All MS/MS samples were analyzed using Mascot (Matrix Science, London, UK; version 2.5.1). The N-terminal sequencing was performed by Edman’s degradation using PPSQ-33A protein sequencing system (Shimadzu Corporation, Kyoto, Japan) at Sangon Biotech (Shanghai) Co., Ltd (Shanghai, China).

### Protease activity assay

Alkaline protease activity was measured by Folin-Ciocalteu method described previously^59^ with slight modification. The suitably diluted enzyme (50.0 μl) was added to 150.0 μl 50.0 mM NaOH-glycine buffer (pH 10.5) containing 2% (w/v) casein and the reaction mixture was incubated at 60 °C for 10 min. The reaction was terminated by adding 200.0 μl 0.4 M trichloroacetic acid (TCA). A blank control was conducted by adding TCA before the enzyme. After centrifuging at 13,000 × *g* for 10 min, 50.0 μl supernatant was pipetted into another tube with 250.0 μl 0.4 M Na_2_CO_3_ and 50.0 μl Folin-Phenol reagent (Shanghai Sangon, Shanghai, China). The mixture was then incubated at 40 °C for 20 min and the absorbance at 680 nm was measured. Tyrosine was used as the standard. One unit (U) of protease activity was defined as the amount of enzyme liberating 1.0 μg of tyrosine per minute at 60 °C under the standard assay conditions. All experiments were repeated three times.

### Effects of pH, temperature, and reagents on enzyme activity and stability

The optimal pH of the purified proteases was assayed at 60 °C in 50.0 mM Tris-HCl buffer (pH 7.0–8.5), 50.0 mM glycine-NaOH buffer (pH 8.5–10.5), and 50.0 mM Na_2_HPO_4_-NaOH buffer (pH 11.0–12.0) containing 2.0% casein (w/v). The optimal temperature was assayed at 40–100 °C for 10 minutes in 50.0 mM glycine-NaOH buffer containing 2.0% casein (w/v) (pH 10.5) with or without 5.0 mM CaCl_2_. The effect of pH on enzyme stability was assayed by incubating enzyme in the previously described buffers^97^ for 2 h at 37 °C. Thermal stability was analyzed by assessing enzyme residual activity after incubating 50.0 μl enzyme solution (0.1 mg ml^−1^) at 60–75 °C with a gradient of 5 °C for 10, 30, 60, and 120 min in 20.0 mM Tris-HCl buffer (pH 8.0) with or without 5.0 mM CaCl_2_.

The effect of metal ions and PMSF on enzyme activity was analyzed by assaying the relative activity with addition of 5.0 mM of metal ions (Na^+^, K^+^, Li + , Ag^+^, Mn^2+^, Zn^2+^, Cu^2+^, Fe^2+^, Ca^2+^, Co^2+^, Ba^2+^, Fe^3+^ and Al^3+^) and PMSF. Similarly, the effect of ethylene diamine tetraacetic acid (EDTA) and ethylene glycol tetraacetic acid (EGTA) on enzyme activity was also analyzed with addition of different concentration (2.0, 5.0 and 10.0 mM). The effect of some surfactants, oxidizing agents and reducing agents on enzyme activity were also analyzed. 1.0% (v/v) of Tween-20, Tween-40, Tween-80, Triton X-100, Triton X-155, JFC-2, β-Mercaptoethanol, Peregal-O and Sodium dodecyl sulfate (SDS) were added for the relative activity evaluation. Meanwhile, different concentration (1.0%, 2.0% and 5.0%, v/v) of H_2_O_2_ was also used for the effect determination on enzyme activity.

### Substrate specificity and kinetic parameters

The substrate specificity of the purified proteases was assayed at pH 10.5 and 60 °C using 1.0% (w/v) substrates, including casein, BSA, gelatin, keratin, and skim milk. The activity toward casein was set as 100%. The kinetic parameters toward the substrates were determined by incubating appropriate diluted enzyme and substrate with concentration from 0.5 to 20.0 mg ml^−1^ at 60 °C for 5 min in 50.0 mM glycine-NaOH buffer (pH 10.5). The *K*_*m*_ and *V*_*max*_ values were calculated by the GraphPad Prism 5.0 software (http://www.graphpad.com/prism/) using non-linear regression. All data are expressed as the means of triplicate measurements.

### Secreted expression in *Bacillus subtilis*

For expression in *B. subtilis*, the AprA-PPC encoding gene with the original signal peptide was codon optimized for *B. subtilis* and synthesized and ligated to pMA5 plasmid (pMA5-AprA-PPC) by TsingKe Biotech Co., Ltd. (Being, China). Besides the original peptide, signal peptides including SP_*lipA*_, SP_*lipB*_, SP_*amyL*_, SP_*amyE*_, SP_*aprE*_, SP_*nprB*_, and SP_*nprE*_ from *B. subtilis* 168 were also used for secreted expression. The expression plasmids containing these signal peptides were constructed by PCR using the modified Gibson assembly method^98^ base on the plasmid pMA5-AprA-PPC. Primers used to amplify the signal peptide fragments for expression plasmid construction were listed in Table S3. The I-5™ 2 × High-Fidelity Master Mix (Tsingke Biotech Co., Ltd, China) was used for PCR amplification. The PCR protocols were as follows: denaturation at 98 °C for 2 min, followed by 30 cycles of denaturation at 98 °C for 20 s, annealing at 55 °C for 20 s, and extension at 72 °C for 3 min, then final extension at 72 °C for 5 min. The plasmid pMA5-AprA-PPC was used as the template. The PCR product was purified by a Cycle-Pure Kit (OMEGA Bio-tek) and then digested by *Dpn*I for 6 h. Then 2 µl of this product and 0.5 µl of Taq DNA ligase were added into 7.5 µl of assembly master mixture^48^, and this mixture was incubated at 50 °C for 1 h. Then the product was directly transformed into competent *E. coli* DH5α and plated onto LB agar plates containing 50.0 μg ml^−1^ of ampicillin and incubated at 37 °C overnight. The positive colony samples were selected to be further validated by sequencing (Tsingke Biotech Co., Ltd, China). All the confirmed recombinant pMA5-AprA-PPC and its derivative plasmids with different signal peptide (Table S2) were than transformed into *B. subtilis* WB600 cells by electroporation to form different recombinant *B. subtilis* WB600 strains for secreted expression. The medium including LB, LBG (LB containing 10% glucose), 2 × LB, 2 × LBG (2 × LB containing 10% glucose), SR (15.0 g l^−1^ tryptone, 25.0 g l^−1^ yeast extract, 3.0 g l^−1^ K_2_HPO_4_) and SRG (SR containing 10% glucose) which containing 60.0 μg ml^−1^ kanamycin and 10.0 μg ml^−1^ chloramphenicol were used for secreted expression. For flask cultivation, 0.5 ml of the seed culture was inoculated into 50.0 ml of these medium in 500 ml flasks and then incubated at 37 °C with 220 rpm.

### Enzymatic dehairing evaluation

Samples of cattle hide, goat, pig and rabbit skins were used for enzymatic dehairing evaluation. The hide and skin samples were cut into appropriate pieces (about 3 × 3 cm) and washed with water several times to remove salt and extraneous matter. An optimized dip method was used for enzymatic dehairing. The dry skin samples were soaked in 10 ml of 50.0 mM NaOH-glycine buffer (pH 9.0) with addition of 0.6 ml 48 h culture supernatant of the recombinant *B. subtilis* WB600A for dehairing treatment at 40 °C for 8–12 h with shaking at 150 rpm. Then the hide and skins were slightly dehaired by rubbing and washing with flowing water. No enzyme addition was used for negative control and the conventional lime-sulfate treatment (by 10.0% lime and 2.0% sodium sulfide based on soaked weight for overnight) was performed as a positive control. The control experiment was also carried out with the same condition as enzymatic treatment. The dehaired hide and skins were evaluated by visual assessment for quality, such as whiteness, softness, hair removal, hair roots and grain surface. The scanning electron microscope (SEM) Hitachi SU8010 (Hitachi, Minato-ku, Tokyo, Japan) was used for these assessments.

### Accession number of sequence in database

The nucleotide sequences of 16 S rRNA gene of *Idiomarina* sp. C9-1 and gene *aprA* have been deposited in the GenBank database under accession number KY855411 and KY887795, respectively.

### Ethical approval

This article does not contain any studies with human participants or animals performed by any of the authors.

## Electronic supplementary material

Supplementary Materials

## Data Availability

The datasets supporting the conclusions of this article are included in the manuscript and additional files.
