# Supplementary material for: A novel alkaline protease from alkaliphilic Idiomarina sp. C9-1 with potential application for eco-friendly enzymatic dehairing in the leather industry
Source: Sci Rep. 2018 Nov 7;8:16467. doi: 10.1038/s41598-018-34416-5 (PMC6220337; doi:10.1038/s41598-018-34416-5)
Supplement: Supplementary file 1 — Supplementary Materials [file 41598_2018_34416_MOESM1_ESM.docx]

**A novel alkaline protease from alkaliphilic *Idiomarina* sp. C9-1 with potential application for eco-friendly enzymatic dehairing in the leather industry**

Cheng Zhou^a*^, Hongliang Qin^a^, Xiujuan Chen^c^, Yan Zhang^a^, Yanfen Xue^a,b^, Yanhe Ma^a,^^b*^

^a^ State Key Laboratory of Microbial Resources, Institute of Microbiology, Chinese Academy of Sciences, Beijing 100101, China

^b^ National Engineering Laboratory for Industrial Enzymes, Institute of Microbiology, Chinese Academy of Sciences, Beijing 100101, China

^c^ School of Pharmaceutical Sciences, Jiangnan University, Wuxi 214122, China

**Supplementary Table S1** Physicochemical properties of some characterized alkaline proteases from different strains.

| **Bacteria strain** | **Optimum** | | **Stability** | | **Specific activity ( U mg^-1^)** |
| --- | --- | --- | --- | --- | --- |
|  | **pH** | **Temp (^o^C)** | **pH** | **Temp (^o^C)** |  |
| *Idiomarina* sp. C9-1 | 10.5 | 60 | 7.0–11.0 | ≤65 | 99511.9 |
| *Bacillus lehensis*^16^ | 12.8 | 50 | 8.0–12.8 | <60 | 29.0 |
| *Bacillus clausii* GMBAE 42^61^ | 11.3 | 60 | 9.0–11.5 | ≤50 | 6852.0 |
| *Thermoactinomyces sp.* HS682^24^ | 11.0 | 50 | 6.0–12.0 | ≤60 | 22.8 |
| *Thermoactinomyces sp.* E79^26^ | 11.0 | 75 | 5.0–12.0 | ≤75 | 580000.0 |
| *Bacillus circulans* M34^59^ | 11.0 | 50 | 8.0–11.0 | ≤40 | 23398.8 |
| *Vibrio metschnikovii* J1^19^ | 11.0 | 60 | 8.0–12.0 | ≤55 | 124040.0 |
| *Bacillus pumilus* CBS^57^ | 10.6 | 65 | 7.0–10.6 | ≤55 | 25500.0 |
| *Termitomyces albuminosus*^54^ | 10.6 | 60 | NA | NA | 184919.8 |
| *Bacillus sp.* NPST‑AK15^51^ | 10.5 | 60 | 7.0–12.0 | ≤50 | 7530.9 |
| *Bacillus* sp. no. AH-101^58^ | 10.5 | 70 | 6.0–12.0 | ≤70 | 2427.0 |
| *Bacillus circulans* MTCC 7942^56^ | 10.0 | 60 | 7.0–12.0 | ≤60 | 24657.7 |
| *Streptomyces koyangensis* TN650^25^ | 10.0 | 70 | 7.0–10.0 | ≤60 | 43750.0 |
| *Caldicoprobacter guelmensis*^65^ | 10.0 | 70 | 7.0–12.0 | ≤80 | 53906.0 |
| *Bacillus pumilus* UN-31-C-42^33^ | 10.0 | 55 | 6.0–11.0 | ≤50 | 17530.0 |
| *Bacillus subtilis* PE-11^60^ | 10.0 | 60 | 8.0–12.0 | ≤60 | 213.6 |
| *Bacillus altitudinis* GVC11^4^ | 9.5 | 45 | 8.0–11.0 | ≤50 | 2061.0 |
| *Virgibacillus sp.* SK37^64^ | 9.5 | 55 | NA | ≤40 | NA |
| *Trametes cingulata strain* CTM10101^52^ | 9.0 | 50 | 7.0–12.0 | ≤70 | 94000.0 |
| *Penicillium chrysogenum* FS010^68^ | 9.0 | 35 | 7.0–10.0 | ≤35 | 36542.6 |
| *Bacillus pumilus* MCAS8^9^ | 9.0 | 60 | 7.0–11.0 | ≤60 | 1960.0 |
| *Alteromonas* sp.^42^ | 9.0 | 45 | 6.0–10.0 | ≤60 | NA |
| *Stenotrophomonas maltophilia* BBE11-1^44^ | 9.0 | 60 | 8.0–12.0 | ≤50 | 82825.0 |
| *Bacillus stearothermophilus* F1^27^ | 9.0 | 70 | 8.0–10.0 | ≤85 | 1790.0 |
| *Aspergillus oryzae*^62^ | 9.0 | 40 | 7.0–10.0 | ≤40 | 92.73 |
| *Hirsutella rhossiliensis*^66^ | 9.0 | 75 | 4.0–11.0 | NA | 139.3 |
| *Scopulariopsis* spp^67^ | 9.0 | 50 | NA | ≤60 | 138.1 |
| *Bacillus licheniformis* A10^8^ | 9.0 | 70 | 7.5–10.5 | ≤65 | 0.082 |
| *Pseudoalteromonas sp.* Strain A28^41^ | 8.8 | 30 | NA*^a^* | ≤68 | 204 |
| *Aspergillus nidulans*^63^ | 8.5 | 40 | 8.0–11.0 | <60 | 5.6 |
| *Thermus sp. strain Rt41A* ^28^ | 8.0 | 60 | 4.5–8.0 | ≤70 | 9370.0 |
| *Bacillus aquimaris* VITP4^15^ | 8.0 | 40 | NA | NA | 1719.0 |
| *Bacillus amyloliquefaciens* SP1^6^ | 8.0 | 60 | 8.0–11.0 | ≤55 | 700.0 |
| *Aspergillus oryzae* CH93^21^ | 8.0 | 50 | 5.0–10.0 | NA | 15.9 |

*^a^* NA means data not available.

**Supplementary Table S2** Plasmids and strains used in this study.

| **Plasmids or strains** | **Features** | **Sources** |
| --- | --- | --- |
| pUC118 | Treated by *Bam*HI and calf intestinal alkaline phosphatase, Amp^R^ | TaKaRa Co., Ltd, China |
| pET28a | Protein expression vector in *E. coli*, Kan^R^ | Merck Co., Germany |
| pET28a-AprA | pET28a derivative with AprA gene | This work |
| pET28a-AprA-PPC | pET28a derivative with AprA gene without PPC domain | This work |
| pMA5 | *E. coli*-*Bacillus* shuttle vector for protein expression in *B. subtilis*, Kan^R^ | BGSC, USA |
| pMA5-AprA-PPC | pMA5 derivative with AprA-PPC gene with original signal peptide, SP*_ori_* | This work |
| pMA5-AprA-PPC1 | pMA5-AprA-PPC derivative, SP*_lipA_* | This work |
| pMA5-AprA-PPC2 | pMA5-AprA-PPC derivative, SP*_lipB_* | This work |
| pMA5-AprA-PPC3 | pMA5-AprA-PPC derivative, SP*_aprE_* | This work |
| pMA5-AprA-PPC4 | pMA5-AprA-PPC derivative, SP*_nprB_* | This work |
| pMA5-AprA-PPC5 | pMA5-AprA-PPC derivative, SP*_nprE_* | This work |
| pMA5-AprA-PPC6 | pMA5-AprA-PPC derivative, SP*_amyE_* | This work |
| pMA5-AprA-PPC7 | pMA5-AprA-PPC derivative, SP*_amyL_* | This work |
| *Idiomarina* sp. C9-1 | DNA source for alkaline protease gene cloning | Laboratory preservation |
| *E. coli* DH5α | Host for gene cloning | Transgen Biotech Co., Ltd, China |
| *E. coli* BL21(DE3) PlysS | Host for protein expression | Transgen Biotech Co., Ltd, China |
| *B. subtilis* WB600 | Host for protein secreted expression, Cm^R^ | Laboratory preservation |
| *B. subtilis* WB600A | WB600 derivative, pMA5-AprA-PPC2, Kan^R^, Cm^R^ | This work |
| *B. subtilis* WB600B | WB600 derivative, pMA5-AprA-PPC7, Kan^R^, Cm^R^ | This work |

**Supplementary Table S3** Primers for expression plasmid construction with different signal peptides by modified Gibson method.

| Primer name | Primer sequences (5’–3’) |
| --- | --- |
| SP*_lipA_*-F | TGTAACAATTTTGATGCTGTCTGTTACATCGCTGTTTGCGTTGCAGCCGTCAGCAAAAGCCGAAACACATCCGAATGAACTGATTCAAACAGAA  CGATGTAACAGACAGCATCAAAATTGTTACAAGTGCAATGATCCTTCTTTTTACAAATTTCATATGTAAATCGCTCCTTTTTAGGTGGCACAAAT |
| SP*_lipA_*-R |  |
| SP*_lipB_*-F | ATTTGTTTATCGCTGATTCTATCTGTTTTAGCCGCTCCGCCGTCTGGCGCAAAAGCTGAAACACATCCGAATGAACTGATTCAAACAGA  GCTAAAACAGATAGAATCAGCGATAAACAAATAATGAATGCCATAAGTACTTTTTTCATATGTAAATCGCTCCTTTTTAGGTGGCACAAA |
| SP*_lipB_*-R |  |
| SP*_aprE_*-F | TTGTTGTTTGCGTTAACGTTAATCTTTACGATGGCGTTCAGCAACATGTCTGCGCAGGCTGAAACACATCCGAATGAACTGATTCAAACAGA  TCGTAAAGATTAACGTTAACGCAAACAACAAGCTGATCCACAATTTTTTGCTTCTCATATGTAAATCGCTCCTTTTTAGGTGGCACAAATG |
| SP*_aprE_*-R |  |
| SP*_amyL_*-F | GGAGCACTAGTGGTGGTGTTGGTTTTGTTTGTATACAGTAGCGGTTTAGCATCTGCAGAAACACATCCGAATGAACTGATTCAAACAGA  CAAACAAAACCAACACCACCACTAGTGCTCCAACCCATTGTAACCGCTTACGATTCATATGTAAATCGCTCCTTTTTAGGTGGCACAAAT |
| SP*_amyL_*-R |  |
| SP*_nprB_*-F | TTACTGGCCGGCTTATGCACAGCGGCCCAAATGGTTTTTGTAACACATGCCTCAGCTGAAACACATCCGAATGAACTGATTCAAACAGAAATTG  CATTTGGGCCGCTGTGCATAAGCCGGCCAGTAATAGAGATGTCTTGGTCAAGTTGCGCATATGTAAATCGCTCCTTTTTAGGTGGCACAAATGT |
| SP*_nprB_*-R |  |
| SP*_nprE_*-F | TTGCTGTCGCTGCTTCGTTTATGAGTTTATCAATCAGCCTGCCAGGTGTTCAGGCTGAAACACATCCGAATGAACTGATTCAAACAGAAATTG  TGATAAACTCATAAACGAAGCAGCGACAGCAACAGACAATTTCTTACCTAAACCCATATGTAAATCGCTCCTTTTTAGGTGGCACAAATGTG |
| SP*_nprE_*-R |  |
| SP*_amyE_*-F | GTTATTCGCTGGATTTTTATTGCTGTTTCATTTGGTTCTGGCAGGACCGGCGGCTGCGAGTGCTGAAACACATCCGAATGAACTGATTCAAACAGAA |
| SP*_amyE_*-R | TGAAACAGCAATAAAAATCCAGCGAATAACGGCAGTAAAGAGGTTTTGAATCGTTTTGCAAACATATGTAAATCGCTCCTTTTTAGGTGGCACAAA |

**Supplementary Fig. S1**


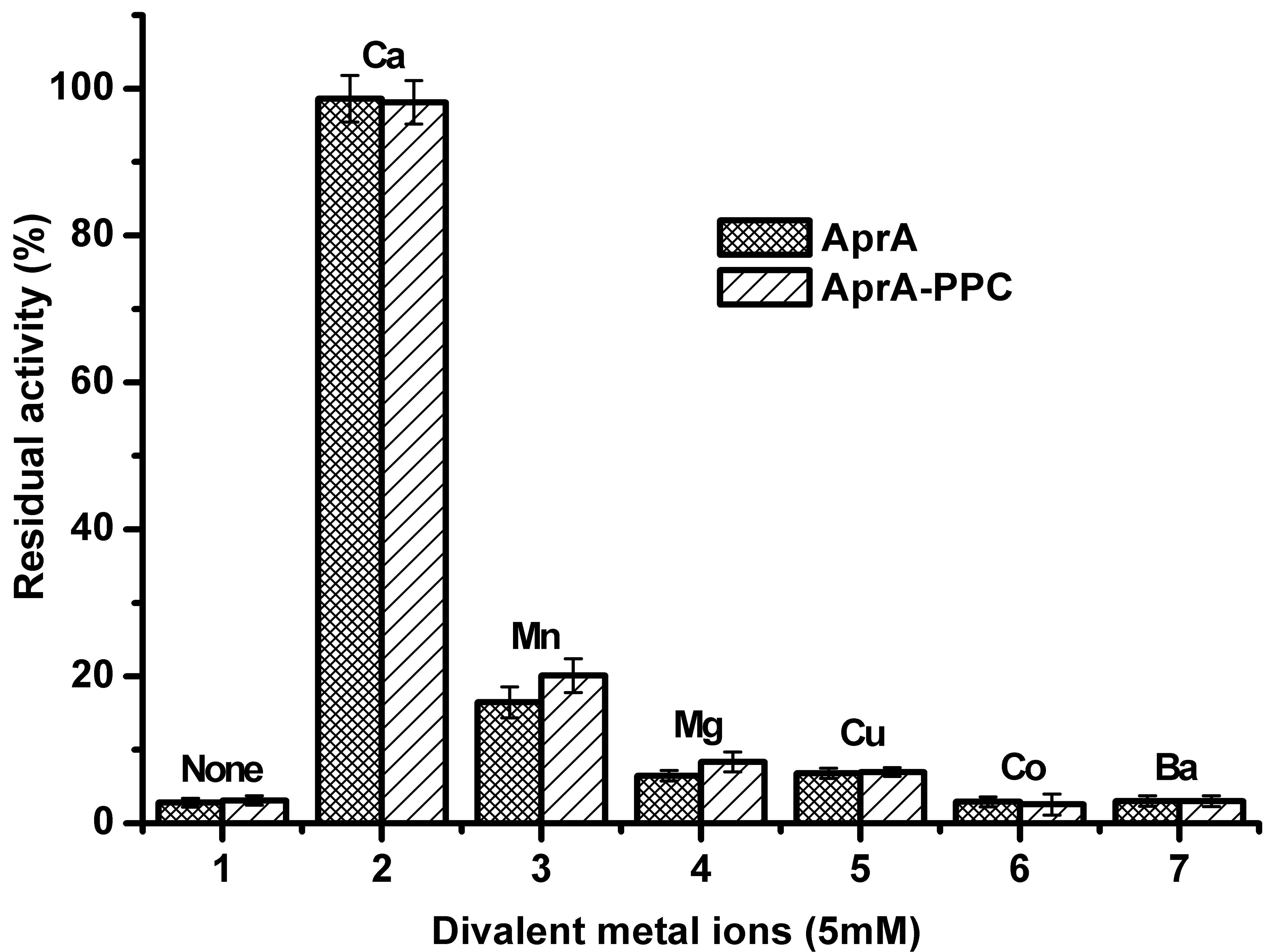


**Fig.S1** The heat/GdnHCl inactivation experiment of AprA and AprA-PPC. The enzyme was dissolved in 20 mM Tris-HCl buffer (pH 7.5) containing 1 M GdnHCl or the same buffer containing 5 mM CaCl_2_, MnCl_2_, MgCl_2_, CuSO_4_, CoCl_2_ and BaCl_2_, respectively. The enzyme solution was incubated at 60^o^C for 10 min and then the residual activity was determined at the standard reaction condition. The activity of the enzyme without incubation was set as 100%. None means without divalent metal ions. The measurements were performed in three independent experiments. Error bars represent standard deviations.
